# Mapping GlycoRNAs on an Exosomal Surface

**DOI:** 10.1021/jacs.5c19319

**Published:** 2026-01-05

**Authors:** Anita Yadav, Anu Sharma, Parmeshwar V. Gavande, Aparajita Nandy, Mohini Moulick, David E. Clemmer, Chandan K. Sen, Subhadip Ghatak

**Affiliations:** † McGowan Institute for Regenerative Medicine, Department of Surgery, 6614 University of Pittsburgh, Pittsburgh, Pennsylvania 15219, United States; ‡ Department of Chemistry, Indiana University, Bloomington, Indiana 47405, United States

## Abstract

This study introduces an innovative periodate oxidation and oxime ligation (OL) method in combination with RNA-binding dyes or molecular beacons (MB) to detect glycoRNAs on endosome-derived exosome surfaces, addressing the challenges posed by exosomes nanoscopic dimensions and the lack of suitable detection techniques. This method enabled the identification of a direct glycan–RNA linkage, potentially advancing our understanding of glycoRNA biology. The method’s specificity and sensitivity were validated using a sequential dual-labeling approach that distinguished exosome-surface-bound glycoRNAs, which was confirmed by flow cytometry and direct stochastic optical reconstruction microscopy (dSTORM). Furthermore, enzymatic treatments with ribonucleases and peptide-*N*-glycosidase F (PNGase F) elucidated the location and stability of glycan modifications, suggesting a significant role of glycoRNAs in cellular uptake for effective intercellular communication. The approach not only bypasses the need for metabolic chemical reporters but also lays the groundwork for exploring unknown glycoRNA sequences and expanding the investigation beyond sialic acids, thereby broadening the scope of epitranscriptomics and providing potential therapeutic insights.

## Graphical abstract

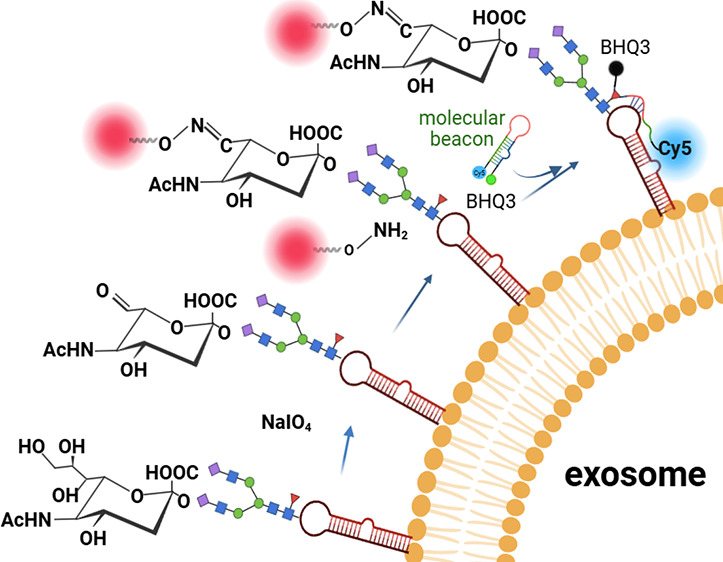

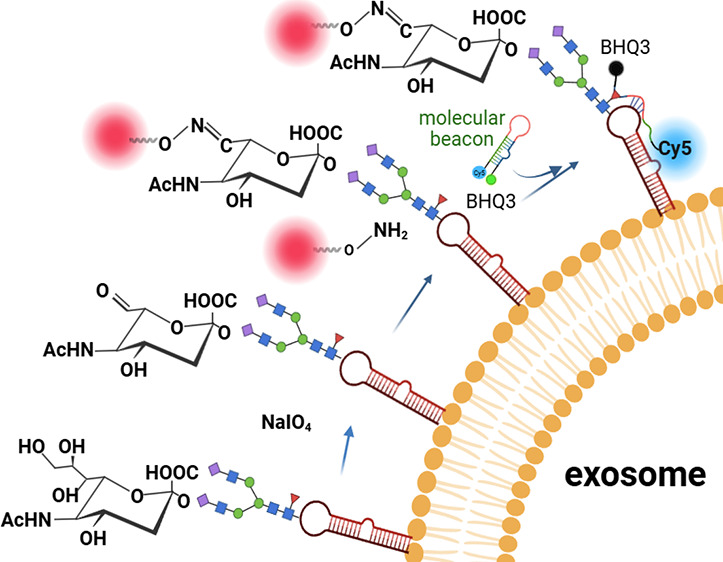

## Introduction

Beyond the traditional function of protein synthesis, ribonucleic acid (RNA) and its modifications have reinvigorated the field of epitranscriptomics.^1,2^ Post-transcriptional modifications of RNA, discovered decades ago, have significantly increased its chemical diversity and functionality, extending beyond its traditional role as a messenger molecule.^3,4^ Despite extensive post-transcriptional RNA modifications found in the epitranscriptome, evidence of carbohydrate linkage was limited to monosaccharide modifications in tRNA only.^5,6^ The recent discovery of RNA glycosylated with sialic acids and fucose on cell surfaces has expanded our understanding of the complexity of RNA modifications in various biological processes, including molecular recognition and cell signaling.^7,8^ GlycoRNAs are small nuclear (sn) RNAs, ribosomal (r) RNAs, small nucleolar (sno) RNAs, transfer (t) RNAs, and Ro-associated Y RNAs, the latter of which comprise the greatest percentage of glycosylated RNA species.^7,9^ Unlike lipids and proteins, RNA was never considered a target for glycosylation.^10^ However, the discovery of glycosylated RNAs (termed glycoRNAs) on cell surfaces supports the notion that RNA can also participate in intercellular communication, a role that was previously thought to be off-limits for RNAs.^10–12^

Our previous work has demonstrated that following injury, bidirectional crosstalk between resident epidermal keratinocytes and blood-borne wound-site macrophages (mΦ) via keratinocyte-originated (Exo_κ_) and mΦ-originated (Exo_mΦ_) exosomes is central for the resolution of inflammation.^13,14^ Compared to uninjured skin, the wound-edge (WE; < 2 mm from the edge) is rich in glycosylated Exo_κ_, which are preferentially internalized by wound-site mΦ via *N*-glycan-mediated uptake mechanisms.^14^ Such Exo_κ_ also showed a high abundance of small bp RNAs (<200 bp) that are critical for the resolution of wound inflammation and restoring the barrier function of the repaired skin.^14^ Current literature indicates that exosomes carry the molecular signature of their parent cells.^15,16^ Unlike ectosomes that originate from the cell membrane^17–20^ and are more likely to carry glycoRNAs on their surface, we tested the hypothesis that RNA molecules might also be anchored on endosome-originated exosomes, expanding beyond the established understanding that RNAs are encapsulated within exosomes. If this hypothesis holds, then it is plausible that these surface RNAs could be glycosylated, akin to glycoRNAs found on cell surfaces.

## Results and Discussion

### Detection of Glycan-Associated Double-Stranded RNA on the Exosome Surface

In this work, exosomes were isolated from the conditioned medium of cultured human keratinocytes using a differential ultracentrifugation method followed by immunomagnetic separation, following MISEV 2023 guidelines,^21^ as previously described by us (EV Track ID: 190103; this method was reported in the EV track that received an EV metric score of 100%).^14^ To ensure specificity, control experiments were conducted in which exosomes were incubated with beads alone and with beads containing aminooxy and TOTO-1 dye. These controls showed no shift in fluorescence, effectively ruling out nonspecific binding with the beads through adsorption (Figure S1A). To investigate the presence of RNA on the exosomal surface, we employed the membrane-permeant SYTO RNASelect Green nucleic acid stain that is selective for RNA and the membrane-impermeant Thiazole Orange Homodimer (TOTO-1) fluorescent RNA-binding stains (Figure 1A). Quantitative bead flow cytometry analysis demonstrated that 15–35% of double-stranded nucleic acids, presumably pre-miRNAs, are localized on the surface of the exosomes (Figure 1B,C). Because TOTO-1 binds to both double-stranded RNA and DNA,^22,23^ it is critical to understand the predominance of both DNA and RNA on the Exo_κ_ surface. DNase I treatment shows a marginal drop in TOTO-1 fluorescence, suggesting the predominance of double-stranded RNA (dsRNA) on the Exo_κ_ surface (Figure S1B–D).

**Figure 1:**
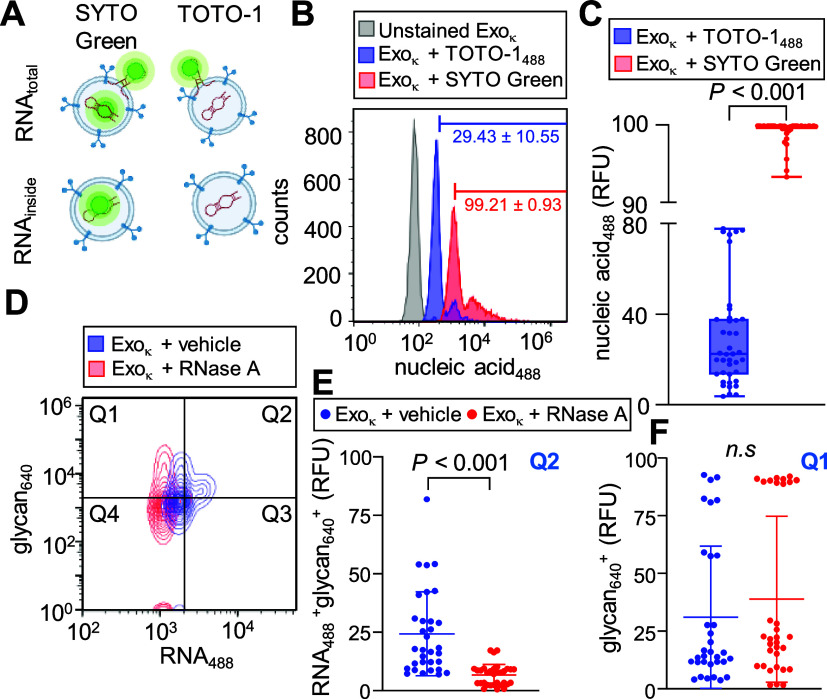
(A) Schematics: SYTO Green and TOTO-1 binding of nucleic acids present on the surface and encapsulated within exosomes (Exo_κ_) isolated from human keratinocytes culture-conditioned media. (B, C) Bead flow cytometric analysis of Exo_κ_ conjugated with super magnetic dynabeads functionalized with CD63, CD9, and CD81, showing the presence of nucleic acid on the Exo_κ_ surface. The histogram demonstrates the shift in FITC (488) fluorescence after binding with SYTO Green and TOTO-1. The mean percentage of beads with exosomes showing FITC fluorescence was plotted graphically (*n* = 30). (D) Representative bead flow cytometry of aminooxy-functionalized 640R (labeled as 640)­Exo_κ_ and TOTO-1 staining with either RNase A or vehicle control. (E) Quantification of glycoRNAs (TOTO-1^+/^OL-640R^+^) and (F) glycans (TOTO-1^–/^OL-640R^+^) after treatment with either RNase A or vehicle control (*n* = 32). Data were shown as mean ± SD and analyzed by Student’s *t*-test for (C) and (E) and Mann–Whitney U nonparametric test in (F). (A) was created with the permission of BioRender.com.

Unlike DNA, RNA’s ribose sugars make it more unstable and susceptible to degradation. Except for long-term RNA persistence (ranging from days to years) reported in various dehydrated tissue types, mainly in the forensic context,^24–31^ RNA stability within the physiological milieu is questionable unless shielded by other biomolecules from endogenous RNase activity. Thus, this observation raises two seminal questions: Are these RNAs on the exosome surface shielded by glycans, forming glycoRNAs? If they indeed exist on the exosome surface, what is their physiological significance in cell–cell crosstalk?

A multitude of methodologies have been utilized to detect and characterize glycoRNAs on cell surfaces.^8,32,33^ Techniques such as click chemistry-based RNA blots and metabolic labeling have confirmed the presence of glycoRNAs on the cell surface.^7^ Further validation has been achieved through imaging and proximity labeling, employing antibodies against dsRNA or glycan-binding proteins such as lectins.^34^ Subsequently, the enrichment of metabolically labeled glycoRNAs using magnetic beads has facilitated their profiling via next-generation sequencing.^35,36^ Concurrently, mass spectrometry coupled with high-performance liquid chromatography (HPLC) has been utilized to elucidate the structure and composition of the attached glycans.^37–39^ Despite substantial advances in LC–MS- and CE–MS-based profiling of cell surface and exosomal *N*-glycans, including recent capillary electrophoresis–mass spectrometry approaches, these methods lack the specificity required to directly assign glycan structures to RNA scaffolds, even though a subset of the detected glycans may plausibly originate from cell- or vesicle-associated miRNAs.^32,40,41^ A novel method, such as ARPLA^32^ (sialic acid aptamer and RNA in situ hybridization-mediated proximity ligation assay), is also of limited use to detect glycoRNA of unknown sequence.^42,43^ This is because ARPLA employs a sialic acid aptamer for glycan recognition and an oligo probe for in situ hybridization of glycoRNAs, which necessitates prior knowledge of the glycoRNA sequences to achieve high selectivity and sensitivity.^42,44–46^ The discovery of glycoRNAs has been significantly advanced using *N*-azidoacetylmannosaminetetraacylated (Ac4ManNAz), a metabolic chemical reporter (MCR) specific for sialic acids.^7,47^ The incorporation of Ac_4_ManNAz leverages the azido group’s reactivity for biorthogonal labeling with copper-free click reagents, integrating into sialic acids during biosynthesis.^48^ While Ac_4_ManNAz has facilitated significant advances in glycoRNAs research, its application is restricted to metabolically active cells capable of incorporating this metabolic reporter during biosynthesis.^49^ This limitation constrains the use of MCRs in translational research, where it is impractical to administer the reporter, as is often the case with clinical samples.^49^

Due to the nanoscopic dimensions of exosomes, the detection of glycoRNAs on their surfaces has remained largely unexplored, hindered by the absence of appropriate tools and visualization techniques.^11^ Exosomes, although originating from endosomes, inherently resemble plasma membranes, suggesting the possibility of functionalizing them via metabolic glycan engineering combined with biorthogonal click chemistry.^50,51^ In this work, we introduce a straightforward, sensitive, and efficient method that employs periodate oxidation and aldehyde ligation to detect glycans on the exosome surface, thereby circumventing the need for MCRs. This method utilizes RNA-binding dyes in combination with glycan labeling to detect the possible presence of RNA modified with glycans (glycoRNAs) on the exosome surface. The 7′-, 8′-diols in sialic acid are particularly reactive, undergoing rapid oxidation to aldehydes at physiological or mild acidic pH in the presence of periodate. These aldehyde groups on the exosome surface were subsequently conjugated to aminooxy-functionalized 640R molecules, forming stable oxime bonds and thereby facilitating the visualization and analysis of glycoRNA profiles on the exosomal membrane.^33^ Such a labeling strategy has no impact on the morphology, surface charge, and size of the exosomes (Figure S2A–C). However, the oxidation of *cis*-diol groups by sodium periodate under mildly acidic conditions poses significant challenges for RNA stability. To assess the impact of sodium periodate treatment on RNA integrity, we designed single-stranded and double-stranded oligonucleotides with identical base sequences (Figure S3A). The integrity of these sequences post-sodium periodate treatment was analyzed using high-resolution automated electrophoresis (Figure S3B). The results demonstrated that while single-stranded RNA (ssRNA) stability was substantially compromised, no changes were observed in the dsRNA (Figure S3C,D). This suggests that the TOTO-1 fluorescence detected after sodium periodate treatment can be attributed to dsRNA, supporting the notion that pre-miRNA is present in abundance on the exosome surface.

The specificity of this two-step glycan tagging approach was further validated by using direct stochastic optical reconstruction microscopy (dSTORM) in combination with the tetraspanin markers CD9, CD63, and CD81. This methodological approach ensures both the specificity of glycan detection and the precise localization of these biomolecules on the exosome surface (Figure S4A–C). Bead flow cytometric analysis using aminooxy-functionalized 640R Exo_κ_ and TOTO-1 demonstrated that 30% of RNA is glycosylated and attached to the exosomal membrane. To assess whether RNA–glycan association within exosomes involves noncovalent interactions, we pretreated the vesicles with guanidine hydrochloride (GdnHCl), a chaotropic agent that disrupts hydrogen bonds, ionic interactions, and hydrophobic associations without cleaving covalent bonds. The persistence of a dual RNA–glycan signal following GdnHCl treatment suggests that the RNA and glycans are linked by covalent bonding (Figure S5A,B). For further rigor, we digested the RNA moiety of glycoRNA at 37 °C with ribonuclease A (RNase A), which selectively cleaves the P–O5′ bond in ssRNA adjacent to pyrimidine residues. RNase A treatment abrogates the glycoRNA signal by ∼75%, leading to the notion that the glycan present on the surface of Exo_κ_ is bound to single-stranded RNA or the loop structure of hairpin RNA (Figure 1D,E). The free glycan signal remained largely unaffected (Figure 1F). Furthermore, the glycoRNA signal also remained unaffected with proteinase K treatment (Figure S5C,D).

The discrepancy in our observations was intriguing, as although the oxidation of cis-diol groups by sodium periodate did not reduce the fluorescence signal, treatment with RNase A resulted in substantial abrogation of the signal. This apparent contradiction necessitated rigorous investigation to confirm the structural orientation of RNA on the exosome surface. To address this, we employed the anti-dsRNA monoclonal J2 antibody, which is considered the gold standard for the detection of dsRNA.^52–55^ Contrary to our hypothesis that no dual-positive exosomes would be observed, our analysis revealed that ∼28% of the total RNA on the exosome surface was positive for both TOTO-1 and J2, while ∼32% was positive for only J2 (Figure S6). From this observation, it is important to consider the underlying nucleic acid architecture. TOTO-1, as an intercalating cyanine dye, binds broadly to base-paired regions within any duplex structure, including stem-loop hairpins, pre-miRNA intermediates, or fully double-stranded RNA (dsRNA). Its fluorescence enhancement reflects the extent of base stacking and helical integrity, rather than sequence or biological origin, and thus serves as a general indicator of double-helical domains within RNA. In contrast, the J2 monoclonal antibody recognizes extended A-form helical dsRNA with a minimum duplex length of ∼40 base pairs, associated with replicative or immunogenic RNA species, and exhibits minimal affinity for short stem-loop or pre-miRNA structures that contain bulges or imperfect pairing. The presence or absence of the overlap between their signals can thus distinguish between structured canonical long dsRNA (TOTO-1^+^/J2^+^) regions or the presence of immunoreactive double-stranded RNA (dsRNA), which is tightly packed A-form dsRNA that is probably protected by a protein or lipid or conformationally inaccessible to dye intercalation (Figure S6).

### Surface Glycosylation of Exosomal Precursor miR-21 Enables Inflammatory Signaling

Recent work by our group and others recognizes miR-21 as a major regulator of mΦ biology and the inflammatory process.^56–60^ Our work was the first to show that exosome-borne miR-21 originating from wound-site keratinocytes is delivered to wound mΦ and helps resolve inflammation.^59^ Keratinocyte-derived exosomes were isolated from day 2 (D2) murine WE tissue and uninjured skin,^61^ which demonstrated a significant abundance of miR-21 in (Exo_κ_) (Figure 2A). However, an important consideration often overlooked in major publications is that several studies report mature miR-21 in exosomes based on RT-qPCR assays that also recognize pre-miR-21. Notably, commercially available miR-21 primers used for miRNA cDNA preparation also amplify the miR-21a-5p region of pre-miR-21. To date, none of these publications has conclusively ruled out the presence of pre-miR-21, thereby potentially confounding their interpretation of mature miR-21 in exosomes. An in-depth analysis of EXOmotif sequences in the precursor (pre) and mature miR-21 (also known as miR-21a-5p) revealed that while mature miR-21 has no EXOmotif sequence, pre-miR-21 does have it in the stem-loop structure, and that justifies exosomal loading (Figure 2B). Furthermore, pre-miR-21 is more stable than mature miR-21 as cargo.^62,63^ Previous studies have documented that on the cell surface, glycoRNAs fractionate exclusively with the small RNA (<200 bp) population only.^7^ Given the presence of EXOmotif sequence in pre-miR-21 that are <200 bp in size, the predominance of glycans on small RNA,^7^ an affinity for the hairpin structure to be glycosylated, and their importance in cell–cell crosstalk, it is plausible that pre-miR-21 with the hairpin structure may be glycosylated on the exosome surface for effective cell–cell communication^5,14^

**Figure 2:**
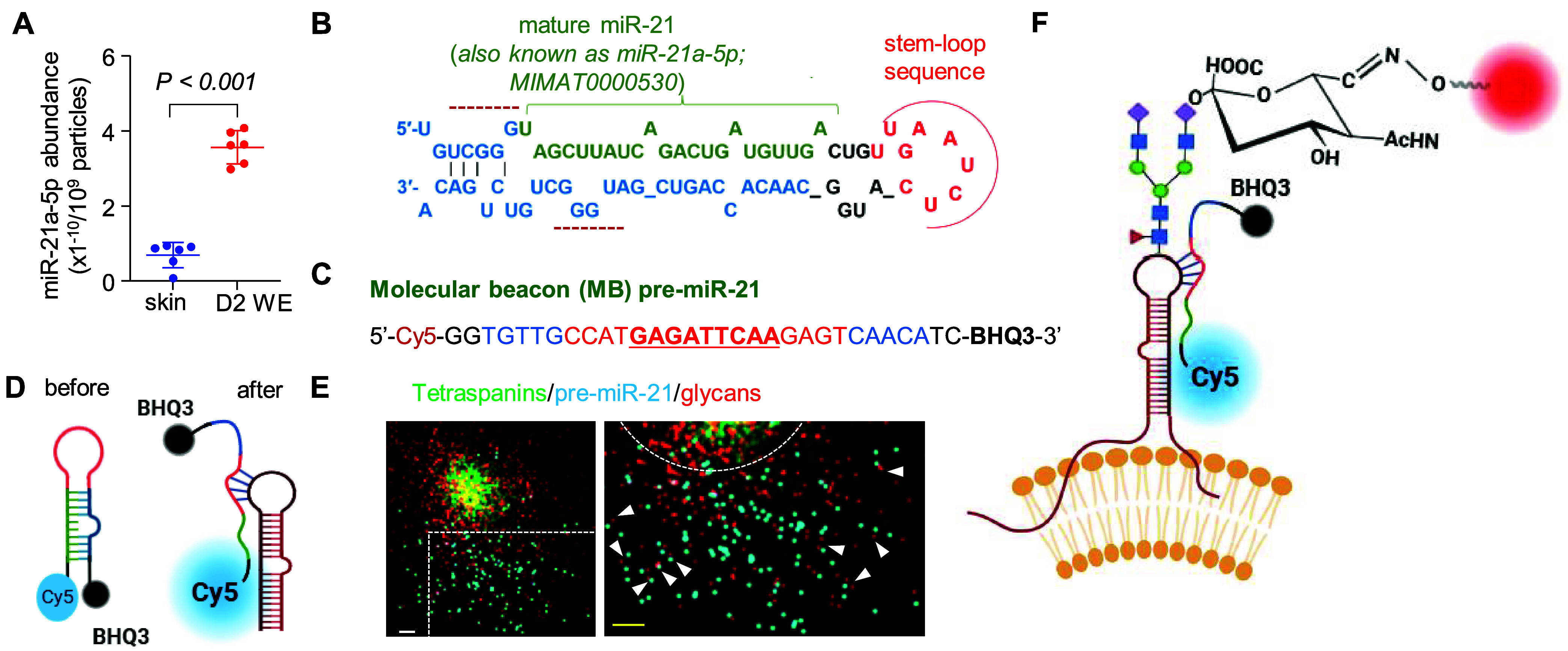
(A) Abundance of miR-21a-5p in murine uninjured skin and D2 WE postwounding shown as mean ± SD and analyzed by Student’s *t*-test. (B) Sequence of pre-miR-21 showing the mature and stem-loop sequence. The red dotted lines indicate the EXOmotif regions. (C) Sequence of MB designed for probing pre-miR-21. (D) Schematics showing the principle of MB fluorescence before and after binding with pre-miR-21. (E) Super-resolution dSTORM images of exosomes isolated from human keratinocytes conditioned media showing localization of tetraspanin markers (CD9, CD63, CD81) on exosomes (green) with glycans (red) and pre-miR-21 (cyan). Scale: 100 nm (white) and 20 nm (yellow). The inset shows the zoomed image of a single exosome with glycans and MB after clustering in CODI software. The white arrowhead indicates glycoRNA as depicted in the schematics. (F) Schematics of glycans (red) and pre-miR-21 (cyan) dSTORM image.

To study the presence of pre-miR-21 on the Exo_κ_ surface, we designed a molecular beacon (MB) with a complementary binding sequence to the hairpin region (Figure 2C). The MB is labeled with Cy5 fluorophores at the 5′-end. The oligonucleotide complementary to the 5′-end of the MB has a black hole quencher 3 (BHQ3) at the 3′-end (designated as the quencher oligonucleotide) and would partially hybridize to form the MB (Figure 2D). This MB design ensures high selectivity for pre-miR-21 compared with mature miR-21. The abundance of glycosylated pre-miR-21 on the surface of murine Exo_κ_ isolated from D2 WE tissue was analyzed using dSTORM nanoimaging with MB in conjunction with glycan labeling. The presence of glycan localization in proximity to the MB signal, distant from the core tetraspanin localization, leads to the notion that the pre-miR-21 on the Exo_κ_ surface is glycosylated (Figures 2E,F and S7). To ensure rigor, the membrane-impermeable nature of the MB was validated using bead flow cytometry (Figure S8A,B). Human keratinocytes were transfected with miR-21-5p by using commercially available XMIR technology (XmiR-21-5p) to package miR-21-5p within exosomes. Transfection with XmiR-21-5p increased the abundance of miR-21-5p in both the cells and the exosomes isolated from the conditioned media of the cultured keratinocytes (Figure S8C,D). Quantitative bead flow cytometry analysis showed no shift in MB fluorescence intensity, demonstrating the impermeable nature of the MB (Figure S8E). Similarly, it is prudent to ensure that RNase activity is entirely a surface phenomenon. RNase A is not naturally membrane-permeable; however, under conditions such as interactions with negatively charged lipid bilayers in malignant cells, modifications such as conjugation with cationic peptides or dimerization can enable it to cross lipid membranes.^55^ Thus, these exosomes transfected with XmiR-21-5p were subjected to RNase A treatment to ensure their membrane-impermeable nature. No change in miR-21-5p transcript abundance was observed (Figure S8F), suggesting that RNase A used in this study does not degrade RNA encapsulated within exosomes.

The sensitivity of this signal was also tested to assess the presence of *N*-linked glycans on exosomal surfaces by using PNGase F. PNGase F cleaves *N*-linked glycans between the innermost *N*-acetylglucosamine (GlcNAc) and asparagine residues of high-mannose, hybrid, and complex glycans from *N*-linked glycoproteins.^64^ This results in a deaminated peptide or protein and a free glycan. We employed PNGase F as a definitive intervention to remove an entire class of glycans, which fundamentally alters antigen expression on exosomes while preserving the vesicle integrity for downstream functional studies. Our findings indicated that PNGase F treatment resulted in the successful digestion of *N*-glycans by ∼60% (Figure S9A). Interestingly, PNGase F treatment also effectively removed glycoRNA signals along with free glycans, indicating a complex linkage between *N*-glycans and RNA (Figure S9B,C). However, the presence of residual glycan signals following PNGase F treatment may be attributed to *O*-glycans, glycoproteins, and glycolipids. For *O*-glycans and glycolipids, more disruptive chemical treatments are necessary that may impact the vesicles integrity. *O*-glycan structural investigations have been challenging due to the absence of a common core structure and a universal enzyme for *O*-glycan release, unlike PNGase F for *N*-glycans. Using enzymatic labeling, we quantified *O*-glycans on exosomal surfaces, finding them to comprise ∼20% (Figure S10). Thus, it is plausible that the residual ∼20% RNA signal may result from RNA bound to *O*-glycans, although the possibility of *O*-glycans bound to protein or other biomolecules on exosomes cannot be ruled out.

### Loss of Exosomal GlycoRNA on Keratinocyte-Derived Exosomes under Diabetic Conditions

Our previous work has demonstrated that Exo_κ_ exhibit functional impairments in diabetes, with surface modifications that reduce their uptake by blood-borne mΦ.^65^ Based on these findings, we hypothesized that Exo_κ_ surface glycosylation/glycation differs under diabetic conditions. To study the distribution and abundance of glycoRNA on Exo_κ_, we isolated Exo_κ_ from the WE tissue of diabetic db/db mice and their heterozygous littermate nondiabetic (m^+^/db) mice D2 postinjury. Unlike nondiabetic m^+^/db mice, the abundance of Exo_κ_ was significantly low in diabetic db/db mice (Figure S11A). The isolated Exo_κ_ from both groups were characterized by their size and surface charges. The Exo_κ_ from diabetic mice exhibited a larger size compared to its littermate nondiabetic control (Figure S11B,C). This was attributed to probable aggregation, as evidenced by multiple peaks in the Nanoparticle Tracking Analysis (NTA). However, the ζ-potential of diabetic Exo_κ_ was significantly higher than that of nondiabetic Exo_κ_, similar to what we observed in human diabetic patients with chronic wounds (Figure S11D).^65^ GlycoRNA abundance was quantified using bead flow cytometry with TOTO-1 dye and an oxime ligation (OL) strategy for glycan labeling. The abundance of glycoRNA was found to be approximately 62% and 7.2% in murine nondiabetic and diabetic Exo_κ_, respectively (Figure 3A,B). We analyzed the distribution of glycosylated pre-miR-21 on the Exo_κ_ surfaces of m+/db and db/db mice. Quantitative bead flow cytometry demonstrated a high abundance of glycosylated pre-miR-21 on the surface of nondiabetic Exo_κ_, unlike diabetic Exo_κ_ (Figure 3C,D). The reduced abundance of glycoRNA on diabetic Exo_κ_ may explain the higher surface charge and decreased uptake by wound mΦ commonly encountered under diabetic conditions. This observation lends credence to our hypothesis that glycoRNAs on the Exo_κ_ surface are the key to effective cell–cell communication, especially keratinocyte-mΦ crosstalk.

**Figure 3:**
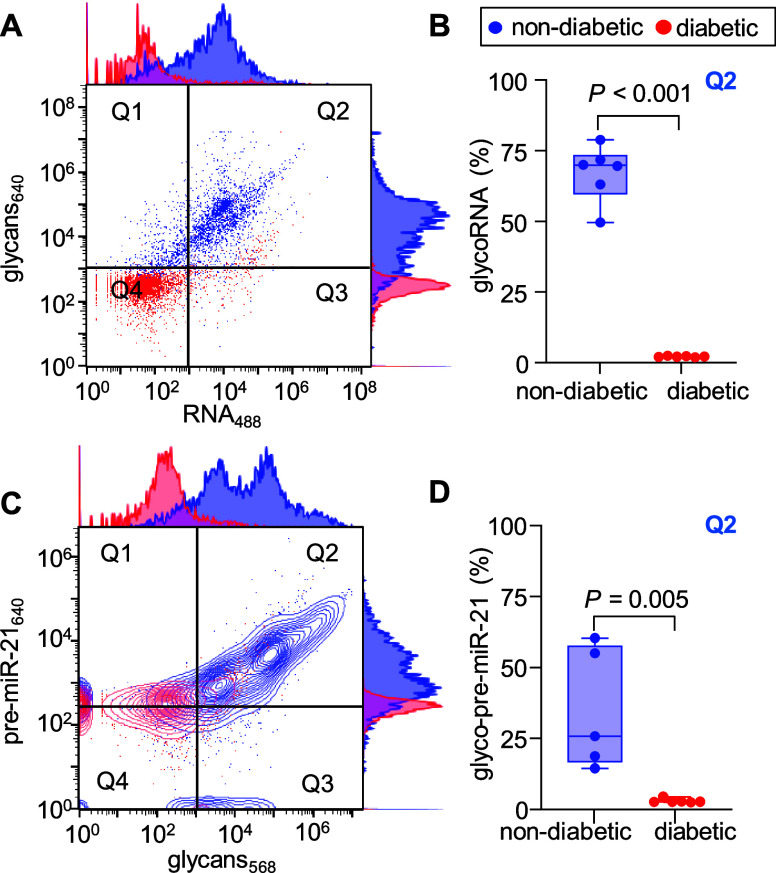
(A) Representative bead flow cytometry of aminooxy-functionalized 640R Exo_κ_ and TOTO-1 staining of Exo_κ_ isolated from D2 WE tissue of nondiabetic m+/db and diabetic db/db mice. (B) Quantification of glycoRNAs (TOTO-1^+/^OL-640R^+^) in m+/db and db/db mice (*n* = 4–6). (C) Representative bead flow cytometry of aminooxy-functionalized 640R Exo_κ_ and MB staining of Exo_κ_ isolated from WE tissue of m+/db and db/db mice. (D) Quantification of glyco-pre-miR-21 (MB^+/^OL^+^) in m+/db and db/db mice (*n* = 5–6). Data in B and D were shown as mean ± SD and analyzed by Student’s *t*-test.

### Surface GlycoRNAs Mediate Exosomal Uptake by Macrophages

Given that glycoRNAs are localized on the Exo_κ_ surface, they are likely to influence exosomal uptake by recipient cells.^64^ Several modes of cellular entry have been proposed for exosomes, and we further investigated the role of glycans as potential determinants of exosomal uptake.^64,66^ Previous studies have demonstrated that glycosidase treatment can alter exosomal surface dynamics by removing glycans, potentially changing the physicochemical properties of exosomes, including ζ-potential^67^ and diameter, and affecting capture in a receptor-independent manner. Conversely, other studies have reported no significant changes in exosome physical characteristics following enzymatic treatment with PNGase F, suggesting that glycans may primarily drive the effects on uptake.^68^ However, vesicle charge was found to be reduced post-treatment, and it remains unclear whether increased uptake is due to glycan–receptor interactions or is related to charge and electrostatic effects. To further explore the role of murine Exo_κ_ surface glycans in cellular uptake, we fluorescently labeled exosomes with the ExoGlow membrane labeling dye following PNGase F treatment. PNGase F treatment does not affect ExoGlow membrane labeling, as demonstrated by bead flow cytometry (Figure S12). The treated and untreated exosomes were then further incubated with murine mΦ, and uptake was assayed via live-cell confocal microscopy (Figures 4A–C and S13). Live-cell imaging revealed compromised uptake of PNGase F-treated exosomes by mΦ compared to that of untreated exosomes. Z-stack analysis revealed that the labeled exosomes were internalized by murine mΦ, with a localization depth of ∼9 μm, confirming their intracellular presence rather than mere surface adhesion (Figures 4C and S13). To further probe the role of exosomal surface RNA in cellular uptake, the exosomes were subjected to RNase, followed by live-cell imaging (Figure S14). Akin to the PNGase F treatment, RNase A-treated exosomes also demonstrated compromised cellular uptake (Figure S15). These findings underscore the pivotal role of glycoRNA on the exosomal surface as a key mediator of cellular internalization.

**Figure 4:**
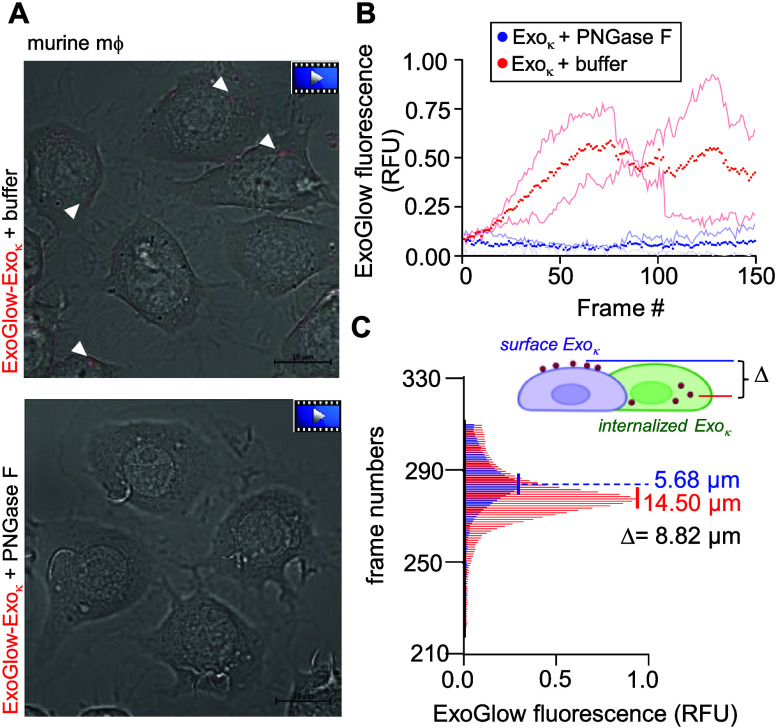
(A) Live-cell confocal images showing compromised uptake of Exo_κ_ by proinflammatory macrophages following the deletion of glycans using PNGase F treatment. Scale: 10 μm. Both Exo_κ_ treated with and without PNGase F were stained with ExoGlow before imaging. (Movie symbol) Indicate movies in the Supporting Information (Videos S1, S2, S3, and S4. S1-PNGase F treated exosomes, S2-PNGase F untreated exosomes, S3-RNase A treated exosomes, S4-RNaseA untreated exosomes). (B) Quantification of the ExoGlow relative fluorescence intensity with time. The replicates are shown in light, and the average is shown in dark-colored lines. (C) Quantification of ExoGlow relative fluorescence from Z-stack images shows that Exo_κ_ with PNGase F treatment fails to enter the proinflammatory macrophages.

### Covalent Glycan–RNA Linkages Define Exosomal Surface GlycoRNAs

A recent study demonstrated that a distinct subset of mammalian small RNAs undergoes *N*-glycosylation at the hyper-modified base 3-(3-amino-3-carboxypropyl)­uridine (acp^3^U), generating surface-exposed glycoRNAs that preserve immune homeostasis.^69^ These glycoRNAs traverse endosomal pathways and appear on the plasma membrane without activating innate sensors. These observations also support the notion that endosome-originated exosomes incorporate glycoRNAs. Furthermore, this also explains our previously published observation that proinflammatory macrophages fail to resolve when the uptake of keratinocyte-derived exosomes is compromised.^13,14,65^ Removal of *N*-glycans by PNGase F unmasks the RNA backbone, converting these molecules into TLR3/7 agonists, while loss of the modifying enzyme DTWD2 abolishes the response.^69^ The glycan thus acts as a structural shield that conceals the immunostimulatory acp^3^U base. Our study demonstrated that, unlike the proximal localization of glycan to the MB signal (Figure 2E), silencing DTWD2 in human keratinocytes using a double nickase plasmid (Figure S16) abrogated the glycan-MB signal, furthermore, suggesting the covalent linkage between the glycans and the pre-miRNA (Figure 5). Collectively, these findings establish RNA *N*-glycosylation as a chemically encoded mechanism of immune evasion and homeostatic resolution, bridging RNA chemistry, glycobiology, and innate immune regulation.

**Figure 5:**
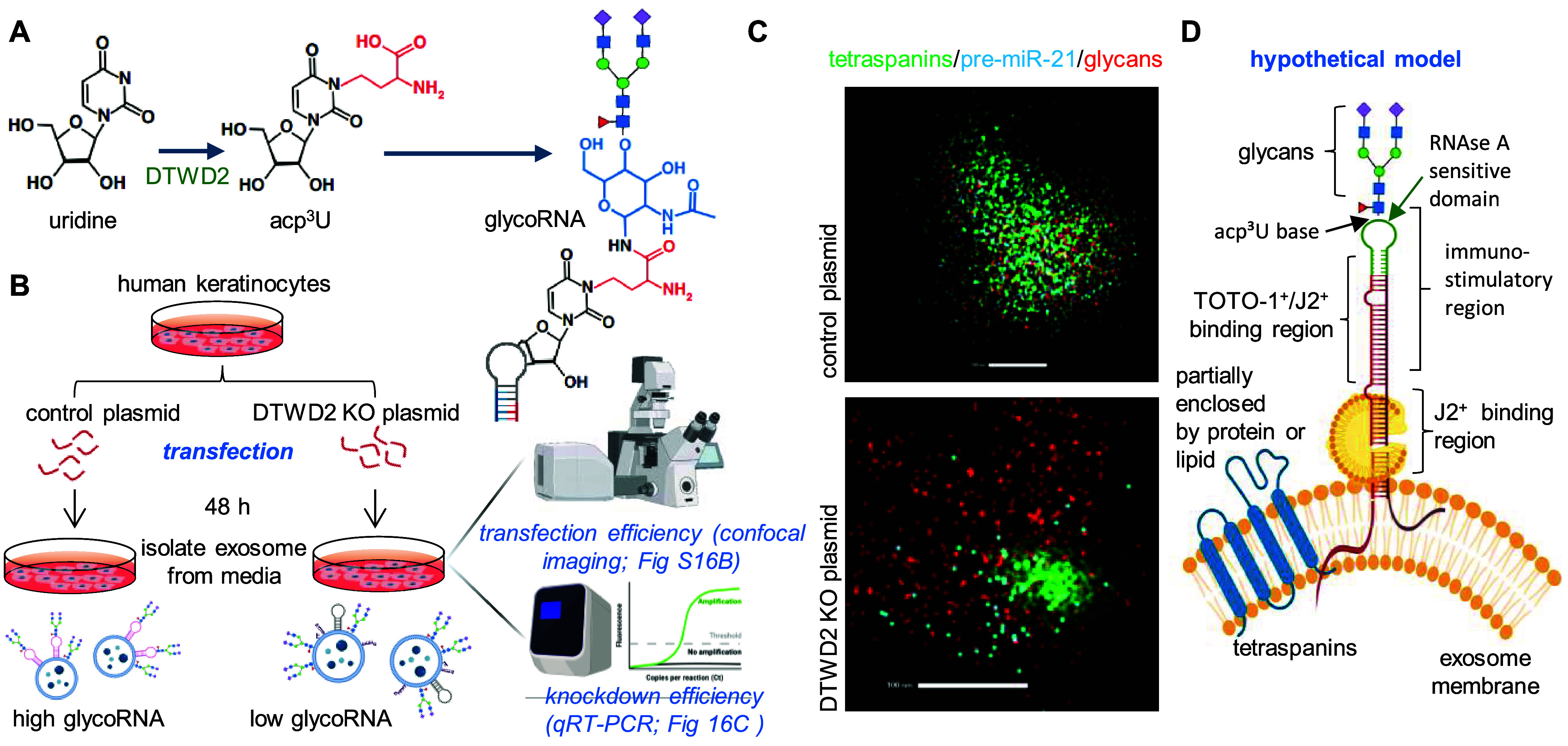
(A) Schematic showing conversion of uridine to the modified RNA base 3-(3-amino-3-carboxypropyl)­uridine (acp^3^U) by DTWD2 as a site of attachment of *N*-glycans in glycoRNA. (B) Schematic showing the knockout of the DTWD2 enzyme using DTWD2 double nickase plasmids. (C) Super-resolution dSTORM images of exosomes isolated from human keratinocyte-conditioned media following transfection by control and DTWD2 double nickase plasmids, showing localization of tetraspanin markers (CD9, CD63, CD81) on exosomes (green) with glycans (red) and pre-miR-21 (cyan). Scale: 100 nm (white) (D) Hypothetical model of glycoRNA on an exosome surface.

Interestingly, another endoribonuclease, MCPIP1 (Zc3h12a), is reported to cleave the terminal loops of pre-miRNAs and counteract Dicer, and primarily inhibits mRNA de novo miRNA biosynthesis (Figure S17A).^70^ Following treatment with MCPIP1 resulted in no change in the glycoRNA signals, suggesting that the cleavage of the terminal loop is not enough to dissociate the glycoRNA signal. This leads to the plausible hypothetical model of glycoRNA on the exosome surface, suggesting that the two strands are either embedded or bound with another biomolecule that prevents the disassociation of the two strands on the exosome surface (Figure S17B,C).

While this manuscript was under revision, a preprint on the bioRxiv server^71^ and subsequently published in PubMed^72^ reported that extracellular exosomal RNAs undergo glyco-modification. These glycoRNAs are not merely surface-associated but are also encapsulated within the exosomes.^71^ However, the subcellular fractionation method used does not conclusively rule out the presence of plasma membrane-derived vesicles.^73–75^ Consequently, the use of CD9, which is also found on the plasma membrane, is insufficient to confirm exosome purity in the absence of specific endosomal markers such as TSG101, Rab5A, or ALIX.^21,76–78^ The use of a precipitation-based exosome isolation method, unlike density gradient ultracentrifugation, without proper size validation, does not rule out the potential contamination from other membrane-originated vesicle types, including microvesicles, apoptotic bodies, and protein aggregates.^79,80^ Although the study relies on GW4869 and Manumycin-A to inhibit exosome biogenesis, it is important to acknowledge that these two inhibitors have a direct bearing on Golgi function and vesicle trafficking,^81^ raising concerns about whether the observed glycoRNA accumulation in the study is solely due to exosome inhibition or whether it also reflects broader disruptions in cellular vesicular transport.

In alignment with the study performed by Flynn et al.,^7^ we conducted click chemistry reactions post-enzymatic treatments with RNase A and PNGase F. We performed both early click and late-click procedures concurrently and observed no significant differences between the two approaches. This aspect of the study further underscores the robustness and reproducibility of the glycoRNA detection methodology under different experimental conditions. The sensitivity and robustness of the OL method and MB approach together have facilitated the discovery of a direct glycan–RNA linkage on the surface of exosomes.^33^ The versatility of this method is anticipated to significantly advance the understanding of glycoRNA biology.

## Conclusions

We posit that this labeling technique will expedite the characterization of the glycoRNA structure and function. Despite these advancements, the biochemical and functional characteristics of glycoRNA remain largely unexplored. There is a critical need to develop imaging techniques for glycoRNAs with unknown sequences, potentially employing general RNA recognition reagents. Furthermore, expanding the labeling and investigation of monosaccharides beyond sialic acid could provide deeper insights into glycoRNA biology. We foresee that applying advanced imaging techniques to visualize glycoRNAs will be a pivotal development in future research. This work provides key insight into the mechanistic underpinnings of impaired uptake of diabetic Exo_κ_, which is known to cause unresolved inflammation and wound chronicity in diabetic patients.

## Supplementary Material











## Notes

PNGase F treated exosomes (Video S1) (AVI)
PNGase F untreated exosomes (Video S2) (AVI)
RNase A treated exosomes (Video S3) (AVI)
RNase A untreated exosomes (Video S4) (AVI)
PDF
