# Supplementary material for: Mapping GlycoRNAs on an Exosomal Surface
Source: J Am Chem Soc. 2026 Jan 5;148(2):2739–48. doi: 10.1021/jacs.5c19319 (PMC12833856; doi:10.1021/jacs.5c19319)
Supplement: Supplementary file 5 [file ja5c19319_si_005.pdf]

Supporting Information

Mapping GlycoRNAs on an Exosomal Surface

Anita Yadav[1†], Anu Sharma[1†], Parmeshwar V. Gavande[1], Aparajita Nandy[1], Mohini Moulick[1], David E. Clemmer[2], Chandan K. Sen[1], Subhadip Ghatak[1*]

[1]*McGowan Institute for Regenerative Medicine, Department of Surgery, University of Pittsburgh, Pittsburgh, Pennsylvania, USA, 15219*

[2]*Department of Chemistry, Indiana University, Bloomington, Indiana, USA, 47405*

[†] *Equal contribution*

Supporting Information

Table of contents

1. Experimental Materials and Procedures

2. Supplementary Figures

Figure S1-Figure S17

3. Supporting References

Supporting Information

1. Experimental Materials and Procedures

Cells and cell culture. The immortalized human keratinocytes (HaCaT) and murine macrophage cell line (RAW 264.7, TIB-71, ATCC) were cultured in Eagle's medium (Life Technologies, Gaithersburg, MD) supplemented with Dulbecco's low-glucose (1 g/L) and Dulbecco's high-glucose (4.5 g/L), respectively, as previously described.[1-2] The cells were cultured in 5% CO_2 incubator with humidified air supplemented with 10% FBS and 1% antibiotic-antimycotic (AA) (Life Technologies). All cells were tested for Mycoplasma contamination using Universal Mycoplasma Detection Kit (ATCC, Catalog Number 30-1012K) as per the manufacturer's instructions.[3] For every experiment involving the isolation or uptake of exosomes, exosome-depleted FBS (ThermoFisher Scientific) was used.

Exosome isolation from cell culture media. Exosomes were isolated from HaCaT cell culture-conditioned media supplemented with 10% Exosome-depleted FBS as described previously.[3-4] Briefly, the conditioned media were centrifuged at 10,000 g for 45 min, and the supernatant was collected. Differential ultracentrifugation (Beckman Coulter Optima Max-XP Ultracentrifuge, rotor TLA120.2) was performed to isolate exosomes from the supernatant, followed by immunomagnetic separation using CD dynabeads as described previously.[4] The exosomes were not eluted from magnetic beads for flow cytometry analysis. Nanoparticle Tracking Analysis (NTA), zeta potential measurements, Transmission Electron Microscopy (TEM), Scanning Electron Microscopy (SEM) imaging, and, for functional assays, exosomes were eluted from the beads using elution buffer (ExoFlow Exosome Elution Buffer, EXOFLOWBUFR-2, System Biosciences).

Mouse model. Male C57BL/6 (strain no. 000664) (8-10 weeks old) and diabetic db/db mice (strain no. 000642) with their heterozygous non-diabetic littermate (m+/db) were purchased from The Jackson Laboratory. All animal research followed protocols authorized by the Division of Laboratory Animal Resources (DLAR) at the University of Pittsburgh. All animals used in this study were ear-tagged and randomly assigned to groups using computer-generated algorithms (www.random.org).

Isolation of murine keratinocyte-derived exosomes from wound-edge tissue. Keratinocyte-derived exosomes (Exo$_{\kappa}$) were isolated from murine wound-edge (WE) tissue after tissue nanotransfection with a cocktail of *Krt14* promoter-driven plasmids encoding murine CD63, CD9, and CD81 with "in frame" GFP reporter with differential centrifugation followed by immunoprecipitation, as previously reported.[4] The isolated exosomes were characterized for their morphology, surface charge, size, and concentration.

Tissue nanotransfection 2.0. *In vivo* TNT was performed as described previously, with a modification in the chip design.[5-7] Briefly, the TNT device was placed directly over the exfoliated dorsal murine skin surface.[4, 8] The cocktail of *Krt14* promoter-driven plasmids encoding murine CD63, CD9, and CD81 with "in frame" GFP reporter was loaded (0.05–0.1 μg/μL) in the reservoir. Pulsed electrical stimulation at 100 V was then applied across the electrodes (10 pulses, 10 ms, I<10 mAmp). As reported, this process of nanotransfection, lasting 0.1 s, does not cause any injury to the tissue.[2, 4-5, 7-13]

Scanning Electron Microscopy (SEM). The morphology of exosomes was examined using an SEM (JEOL 7800F, JEOL Japan). Following ultracentrifugation, the exosome pellet was resuspended in 10% glutaraldehyde for 10 min at room temperature (RT). Fixed samples were

drop cast onto stubs with glass coverslips placed on carbon tape and then cured in a vacuum environment for at least 12 h before analysis. The morphology of exosomes was studied by capturing photographs following gold sputter coating at a beam energy of 10 kV.[3]

Zeta potential analysis. The surface charge (zeta potential) of labeled and unlabeled exosomes was measured by diluting the exosome suspension in particle-free water (1:20) using a Zetasizer (Nano-Z, Malvern Instruments Ltd., UK), as previously described.[3-4] The samples were examined using the volume-weighted size distribution mode.

Nanoparticle Tracking Analysis (NTA). The average size and concentration of labeled and unlabeled exosomes were determined using NTA (Nanosight Pro) with a 532 nm laser (Malvern, Worcestershire, UK). All measurements were taken using a sample dilution of 1:100-1:1000. Before measuring, the instrument was calibrated in Milli-Q using 100 nm standard latex spheres (dilution 1:1000). As previously reported, data were analyzed using Malvern Instruments NTA 3.0 software.[3-4]

Periodate oxidation and oxime ligation. For labeling exosomal surface glycans, glycan-containing biomolecules were oxidized to aldehydes before labeling. Exosome-conjugated beads were distributed in 500 μL of PBS and 50 μL of 10X reaction buffer (1 M sodium acetate (CH_3COONa), 1.5 M sodium chloride (NaCl) in ultra-purified water, pH 5.5), followed by the addition of 20 μL of 100 mM sodium periodate ($NaIO_4$) solution (prepared in ultra-purified water). The periodate was allowed to react at RT for 10 min to accomplish oxidation. The periodate was then quenched by adding ethylene glycol $(CH_2OH)_2$ at a final concentration of 100 mM (5 μL of 14.5 M) and incubating the reaction mixture at RT for 10 min. The aminoxy 640R (or aminoxy 568 in accordance with combinations of RNA dyes) dye (aldehyde reactive probe) was employed

at a concentration of 250 nmol (0.5 μL of a 5 mM stock solution prepared in Dimethyl sulfoxide (DMSO)). The oxime ligation was initiated by adding 15 μL of aniline acetate (91057, Biotium). The reaction mixture was left to react at 37 ˚C for 2 h. After the labeling reaction, the labeled exosomes were washed three times with ultra-purified water, and the exosome-conjugated beads were magnetically separated from the unreacted dyes. All stock solutions used in this reaction were made fresh within 20 min of usage.[14]

High-resolution automated electrophoresis of RNA. The integrity of ssRNA and dsRNA and their concentration after sodium periodate treatment were analyzed in an Agilent 2100 Bioanalyzer (Agilent Technologies, Santa Clara, California).[4, 15]

***O*-glycan labeling.** *O*-glycans on exosomal surface were specifically labeled using *O*-glycan labeling kit (EA007, R&D Systems, Inc), as per manufacturer's instructions. Briefly, exosome-conjugated beads were distributed in 500 μL of PBS, and 5 μL of CMP-Cy3-Neu5Ac (0.2 mM), 5 μL of ST3Gal2 (0.2 μg/μL, 7275-GT, R&D Systems, Inc), and 1X Assay Buffer were added to a total reaction volume of 100 μL. The reaction mixture was incubated at 37 °C for 1 h. After the labeling reaction, the labeled exosomes were washed three times with ultra-purified water, and the exosome-conjugated beads were magnetically separated from the unreacted moieties. The abundance of *O*-glycans on the exosomal surface was assessed using bead flow cytometry.

direct Stochastic Optical Reconstruction Microscopy (dSTORM) imaging. Glycans labeled exosomes were analyzed using a 100X oil immersion objective on a Nanoimager S Mark II microscope from ONI (Oxford Nanoimaging, Oxford, UK).[16] Lasers used included 405 nm/150 mW, 488 nm/1 W, 560 nm/500 mW, and 640 nm/1 W. Fluorescence from 488 nm, 560 nm, and 640 nm lasers was recorded using bandpass filters at 498-551 nm, 576-620 nm, and 665-705 nm,

respectively. Glycans labeled exosomes were incubated overnight at 4 °C with anti-Alexa Fluor conjugated anti-CD9, anti-CD63, and anti-CD81 antibodies using the ONI EV Profiler Kit (EV-MAN-1.0, Oxford Nanoimaging, Oxford, UK) according to the manufacturer's instructions. The samples were imaged using freshly generated phosphate-buffered saline (PBS) buffer for glycans and ONI BCubed dSTORM Imaging Buffer for detecting tetraspanins. Two channels of dSTORM data (2000 frames per channel) were captured sequentially at 30 Hz in total internal reflection fluorescence (TIRF) mode, with laser powers of 35% for the 640 nm laser and 40% for the 488 nm laser. Before each imaging session, bead slide calibration was done to align fluorescent channels, resulting in a channel mapping precision of less than 12 nm.[3]

Designing a Molecular Beacon (MB). Molecular beacons complementary to the hairpin loop region of pre-miR-21 were designed based on the miRbase sequence and synthesized by Integrated DNA Technologies (IDT). These molecular beacons, which target the loop nucleotide sequences of pre-miR-21, were labeled with Cy5 fluorophores at the 5' end and BHQ3 at the 3' end. All synthesized beacons were suspended in nuclease-free water (1 µg/µL), stored in opaque tubes wrapped in foil, and kept at -80 ºC.

Transfection of human keratinocytes. Human keratinocytes (HaCaT cells) were transfected with either XmiR-21 (100 nM) (SBI Biosciences) and its respective control using DharmaFECT 1 or with CRISPR Cas9 double nickase plasmids system (Santa Cruz Biotechnology Inc., USA, with gene encoding CRISPR Cas9 double nickase D10A, scramble gRNA as negative control, hAhB gRNA pair or h2Ah2B gRNA pair) using Lipofectamine 3000 transfection reagent, as mentioned previously.[4] After 48 h, the transfected cells and conditioned media were collected. Exosomes were

isolated from conditioned media, and the binding of MBCy5 on the exosomal surface was assessed using flow cytometry.

Genomic DNA Extraction and Gap-qPCR. Following site-specific disruption of the DTWD2 locus using a CRISPR–Cas9 double-nickase plasmid, genomic DNA was isolated from human keratinocytes post-transfection using a Genomic DNA Purification Kit (Thermo Fisher Scientific, cat. no. K0512) according to the manufacturer's protocol. Genomic DNA concentration and purity were determined spectrophotometrically using a NanoDrop instrument (Thermo Fisher Scientific) by measuring absorbance at 260 nm. Targeted disruption of the *DTWD2* locus was assessed by PCR amplification of the genomic region flanking the predicted double-nickase cleavage site. PCR was performed using locus-specific primers designed to span the paired gRNA target region.

RNA extraction and quantitative real-time PCR. The RNA from the exosome or human keratinocytes was extracted using the miRVana miRNA isolation kit (Ambion) according to the manufacturer's protocol.[17-18] To determine the expression of miR-21, specific TaqMan assays for miR-21 were utilized in conjunction with the TaqMan miRNA Reverse Transcription Kit. Knockout (KO) efficiency for the DTWD2 gene using the CRISPR Cas9 double nickase system; cDNA synthesis from total isolated RNA was performed using the SuperScript™ VILO™ cDNA Synthesis Kit (Thermo Fisher Scientific, USA). Real-time PCR was subsequently performed using the Universal PCR Master Mix (Applied Biosystems, Foster City, CA) or PowerUP™ SYBR™n Green Master Mix (Applied Biosystems) on Quantstudio 3 (Applied Biosystems).

Flow cytometry analysis. Exosomal RNA was accessed by incubating RNA binding dye SYTO^{TM} RNA select Green (2 µL of 5 mM in DMSO, S32703, ThermoFisher Scientific) and TOTO-1 (2 µL of 1 mM in DMSO, T3600, ThermoFisher Scientific) with the exosomes conjugated with beads

at 37 ˚C for 30 min. For accessing glycoRNA, glycan labeling was performed as mentioned above, followed by the addition of TOTO-1 at the noted concentration and incubated at 37 ˚C for 30 min. For murine Exo_{κ}, MB_{Cy5} (2 µL of 100 nmol, IDT) was incubated with exosomes conjugated GFP beads for 30 min at 37 ˚C. The MB_{Cy5} was preheated at 37 ˚C for 10 min to open the hairpin structure. For analysis of dsRNA on exosomal surface, J2 monoclonal antibody (NBP3-11395, Novus Biologicals) was conjugated with CF647 kit (92449, Biotium) as per manufacturer's instructions and used at a dilution of 1:200. Additionally, the samples with and without PNGase F/RNase A treatment were labeled with ExoGlow™-Membrane EV Labeling Kit (EXOGM600A-1, System Biosciences) as per manufacturer's instructions to study the effect of deglycosylation.[19]

All the tagged exosomes were subsequently purified by washing them thrice with ultra-purified water, followed by magnetic separation of the exosome-containing beads from the unreacted dyes. The samples were analyzed using an Accuri C6 flow cytometer (Accuri Cytometers, Michigan, USA). Data from 5000 to 10,000 events were gathered at a rate of 250-300 events/s and analyzed with FlowJo software (Tree Star, OR, USA). FITC, PE, and APC fluorescence was determined using FITC, PE, and APC channels.[20] For the rigor and control set, beads were incubated with different dyes under similar conditions.

Enzymatic treatment. The RNA and DNA moiety of glycoRNAs was digested by adding RNase A (20 µL of 1 mg/mL, AM2271, Thermofisher Scientific) and DNase I (1 U of 1U/ µL, EN052, Thermo Scientific) to the exosomes conjugated with beads and incubated at 37 °C for 30 min. The glycan moiety was digested by adding PNGase F (5 µL of 500,000 units/mL, P0704, New England Biolabs) to the samples and incubating at 37 °C for 16-20 h. Glycan labeling was performed on the treated and untreated samples, followed by the addition of TOTO-1.

Exosome uptake assay. For cellular uptake of Exo_{κ} (with and without PNGase F/RNase A treatment) by murine macrophages, exosome concentration was measured by NTA and labeled with ExoGlow™-Membrane EV Labeling Kit. The ExoGlow™-labelled exosomes (10^8 particles) were added to the murine macrophages (RAW 264.7 cells, pretreated with LPS (1 µg/mL) for 24 h), and live-cell imaging was performed using the LSM 880 confocal microscope (Zeiss) as described previously.[3-4]

2. Supplementary Figures

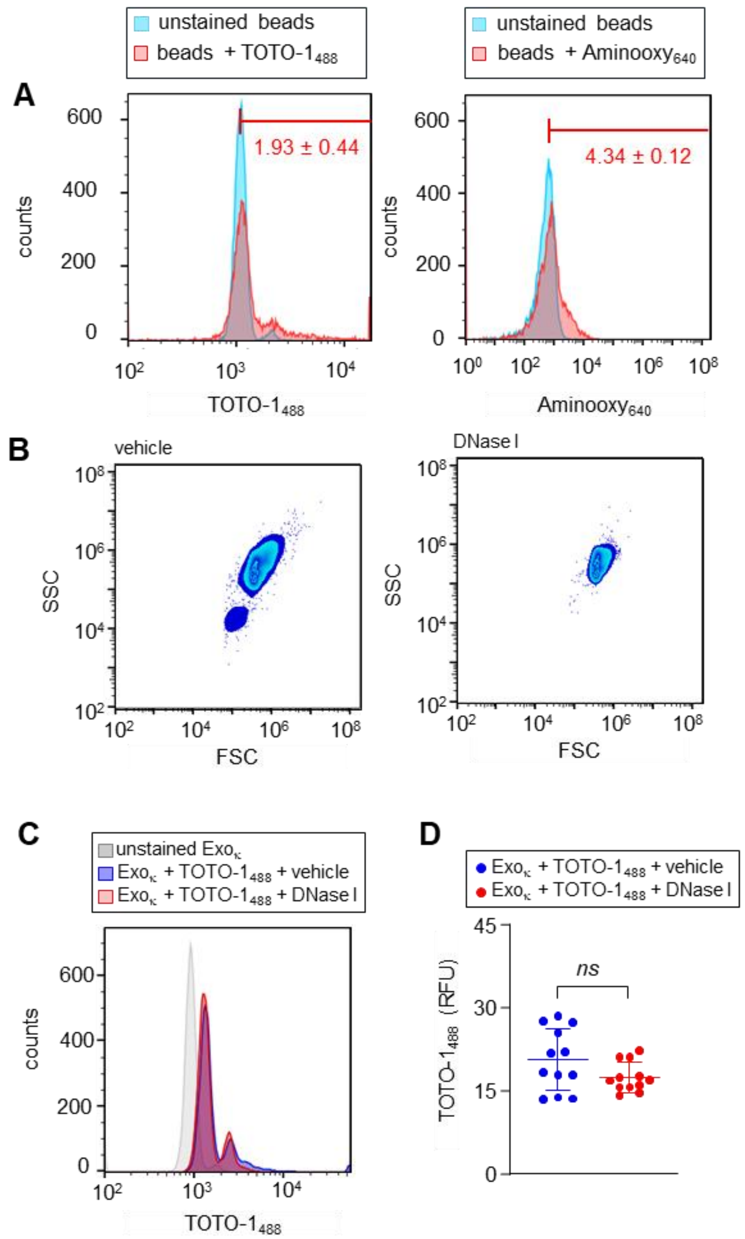

Figure S1. (A) Representative beads flow cytometry histogram plot showing TOTO-1 and aminooxy dye fluorescence of super magnetic dynabeads^{TM} functionalized with CD63, CD9, and CD81. (n=3). Data shown as mean ± SD. (B) Representative beads flow cytometry scatter plot showing TOTO-1 fluorescence of Exo$_k$ treated with either vehicle or DNase I. (C-D) Beads flow

cytometric analysis of Exo$_\kappa$ conjugated with super magnetic dynabeads^{TM} functionalized with CD63, CD9, and CD81 showing presence of RNA on Exo$_\kappa$ surface. The histogram shows the shift in FITC fluorescence upon binding to TOTO-1. The mean percentage of beads with exosomes showing FITC fluorescence was plotted graphically. (n=30). Data in (C) are shown as mean ± SD and analyzed by Student's t-test.

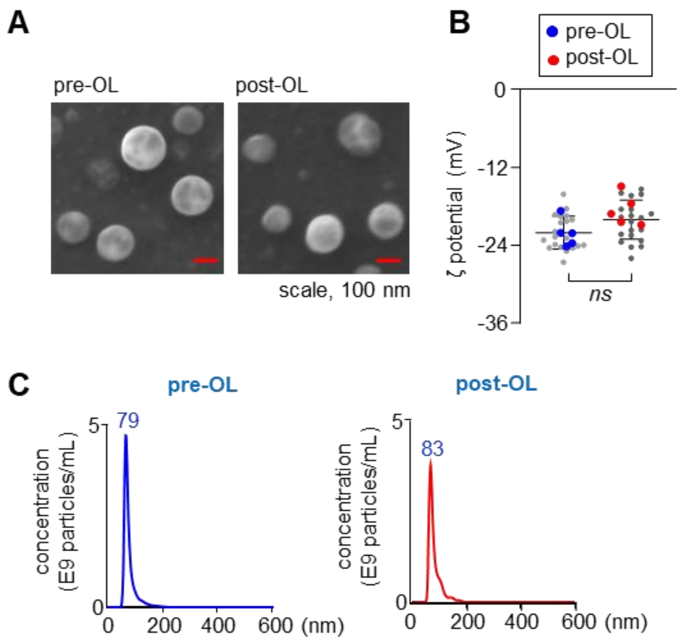

Figure S2. Oxime ligation (OL) approach for detecting the presence of glycans on the exosome surface has no significant effect on (A) morphology as observed by scanning electron microscope, (B) zeta potential, and (C) size distribution as observed by Nanoparticle tracking analysis. In B, each dot corresponds to one technical replicate, except the blue and red dots, which correspond to the mean of biological replicates. At least 4 technical replicates per sample. (n=5). Data in (B) is shown as mean ± SD and analyzed by Student's t-test.

A ssRNA sequence: GCGACAGAUGCAAUUUUUGUACUACACAAAAGUACUGGUCAUUUAAGUU

No of bases: 49

dsRNA sequence: GCGACAGAUGCAAUUUUUGUACUACACAAAAGUACUGGUCAUUUAAGUU
CGCUGUCUACGUUAAAAACAUGAUGUGUUUUCAUGACCAGUAAAUUCAA

No of bases: 98

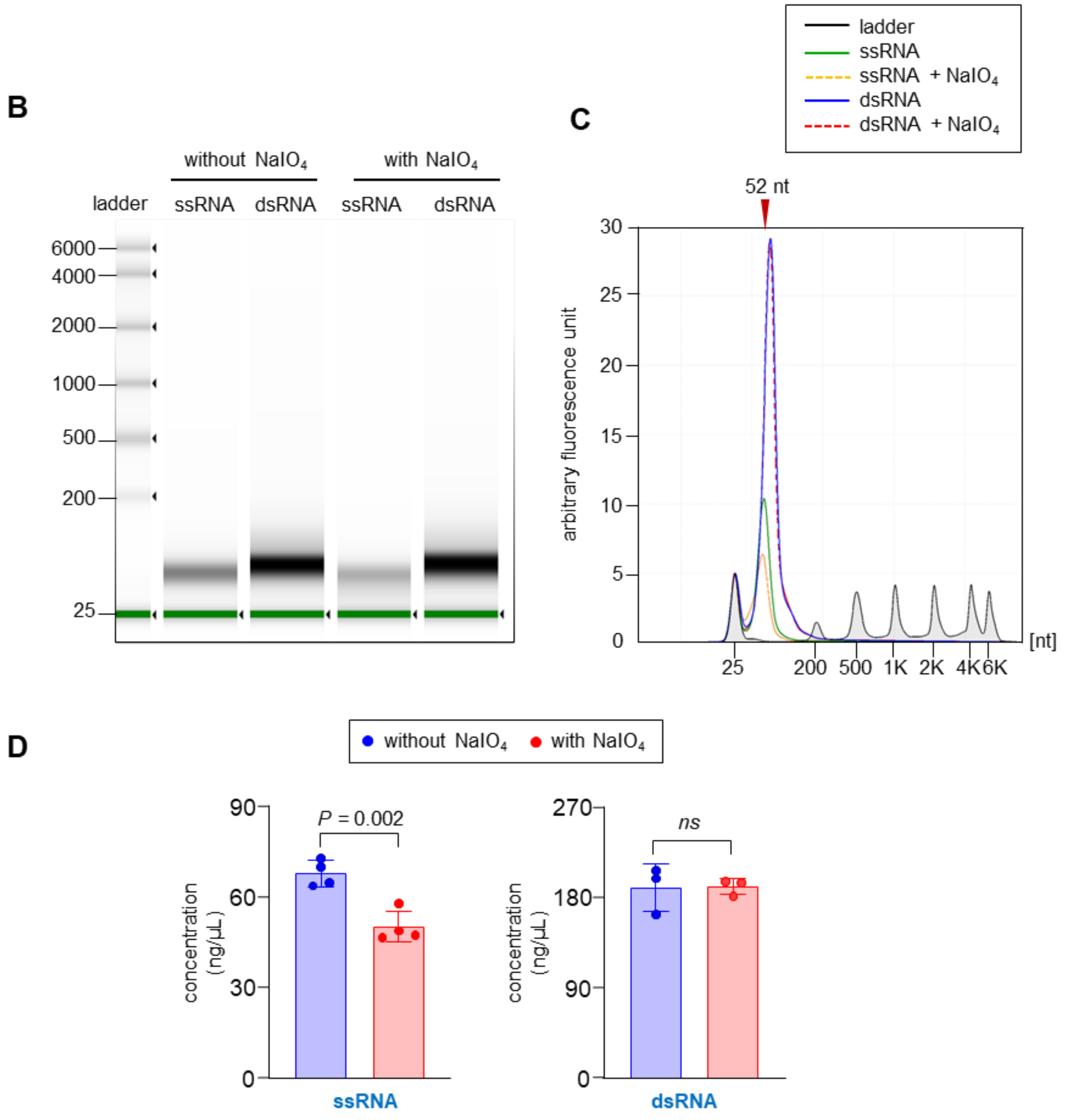

Figure S3. Effect of sodium periodate ($NaIO_4$) treatment on the integrity of RNA. (A) Sequence of single-stranded (ssRNA) and double-stranded RNA (dsRNA). (B) High-resolution automated electrophoresis of ssRNA and dsRNA shows no effect on the integrity of dsRNA but a significant effect on the integrity of ssRNA. (C) Comparison of bioanalyzer-generated electrophoretograms of ssRNA and dsRNA. (D) Comparison of concentration (ng/µL) of ssRNA and dsRNA before and after sodium periodate treatment. (n=3-4). Data in (D) is shown as mean ± SD and analyzed by Student's t-test.

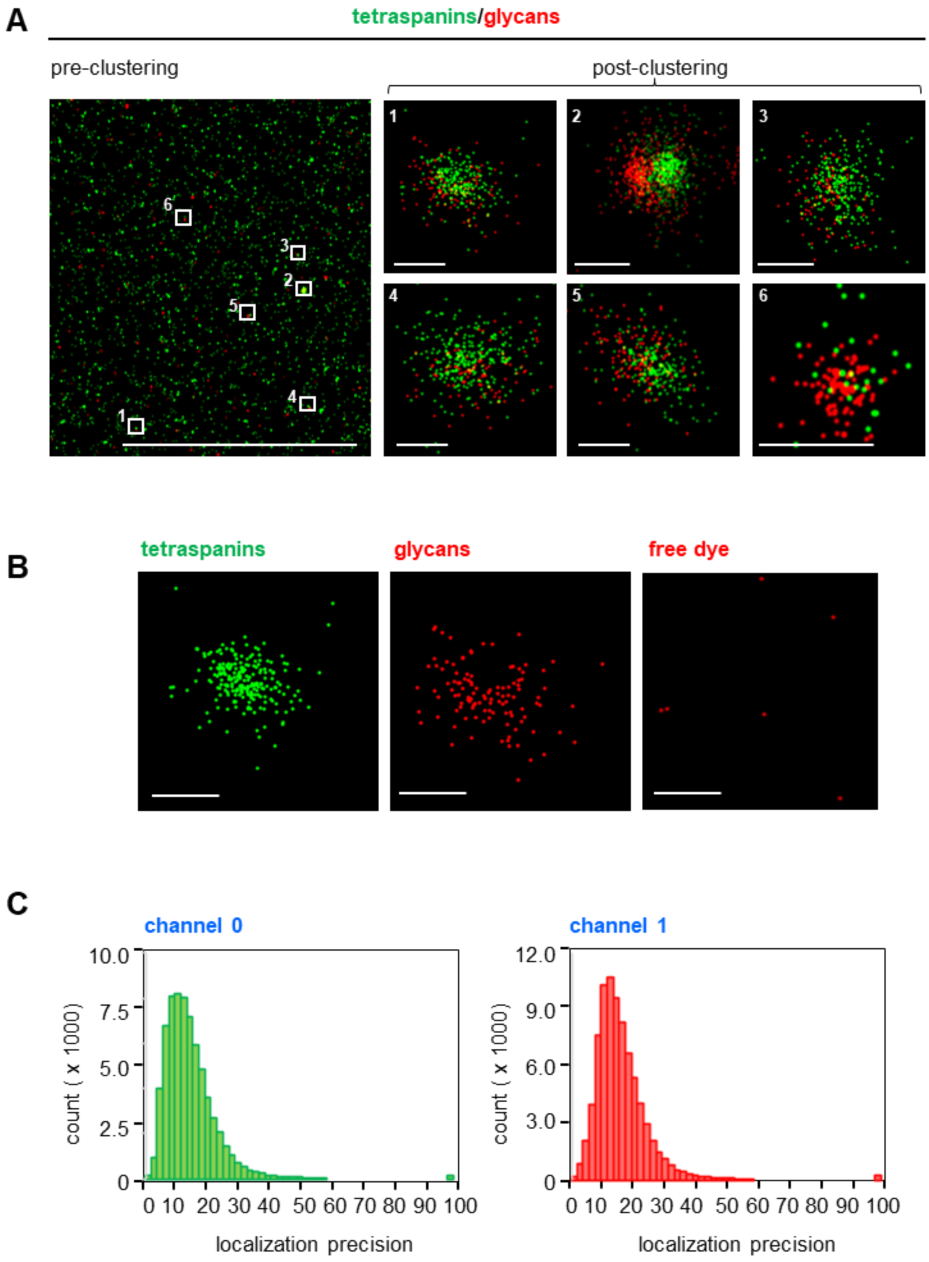

Figure S4. (A) Super-resolution dSTORM images of exosomes isolated from human keratinocytes conditioned media showing localization of tetraspanin markers (CD9, CD63, CD81) on exosomes (green) and glycans (red). Scale, 10 µm. Inset showing several zoomed images of a single exosome with glycans after clustering in CODI software. Scale, 100 nm. (B) Super-resolution dSTORM single-channel image of tetraspanins, glycans, and CF free dye molecules used for oxime ligation

after clustering in CODI software. Scale, 100 nm. (C) Localization precision of the individual channel 0 (tetraspanins) and channel 1 (glycans).

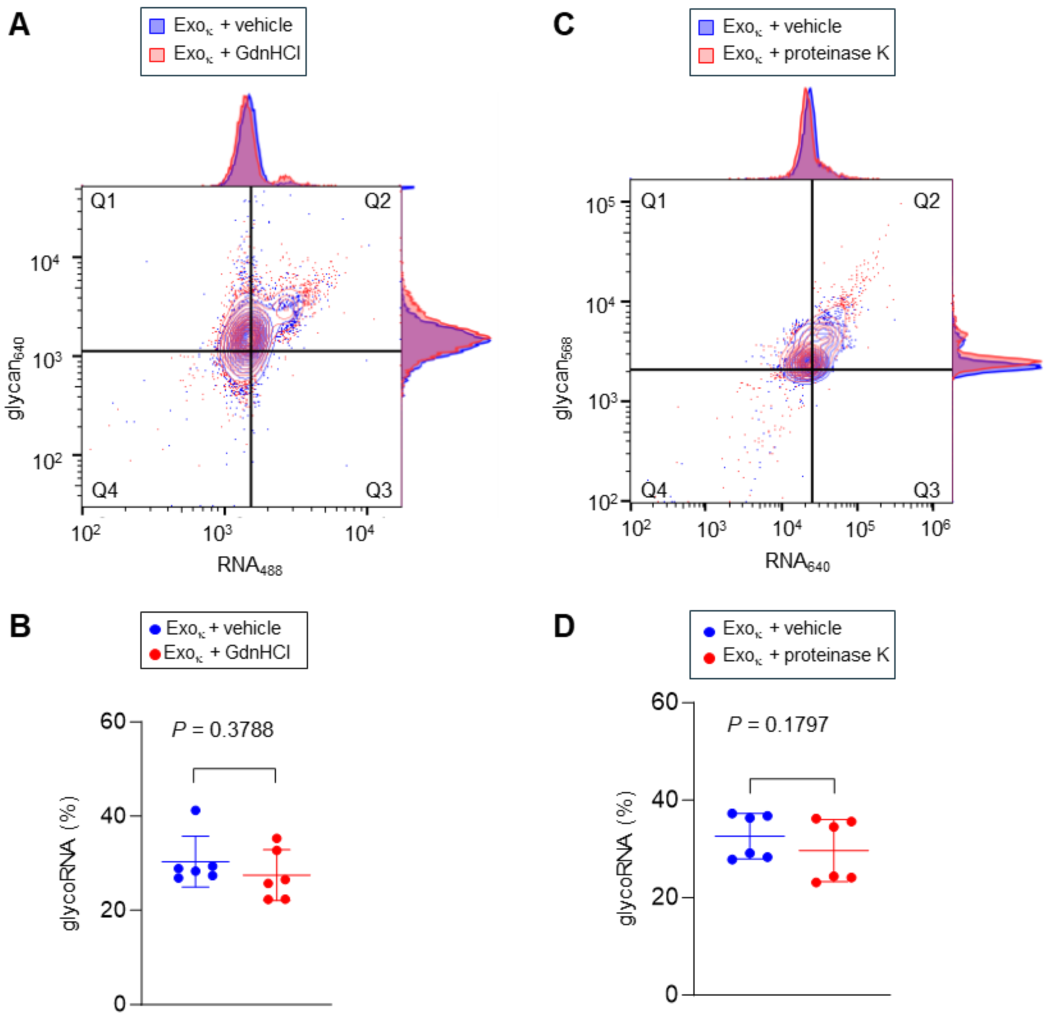

Figure S5. Beads flow cytometric analysis of Exoκ conjugated with super magnetic dynabeads™ functionalized with CD63, CD9, and CD81, showing the presence of glycoRNA with either GdnHCl or its respective control (A,B) or either proteinase K or its control (C,D) on Exoκ surface. Data shown as mean ± SD and analyzed by Student's t-test for (B) and Mann-Whitney U non-parametric test in (D). (n=6)

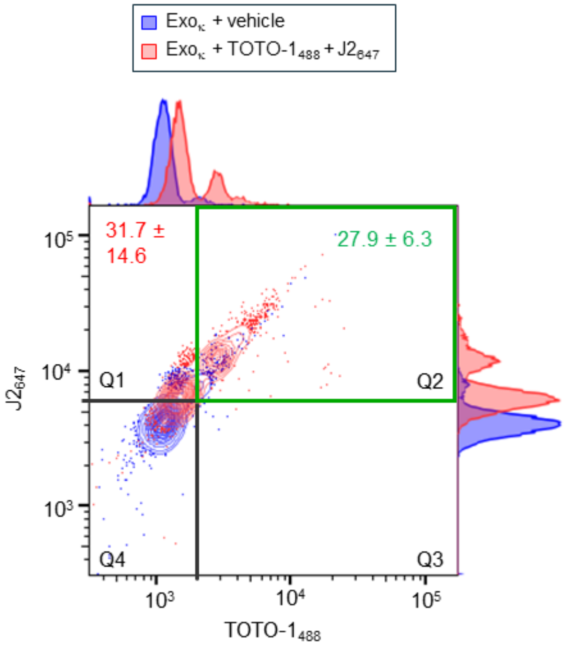

Figure S6. Beads flow cytometric analysis of Exoκ conjugated with super magnetic dynabeads™ functionalized with CD63, CD9, and CD81, showing the presence of both ssRNA as well as dsRNA on Exoκ surface. Data shown as mean ± SD and analyzed by Student's t-test. (n=9)

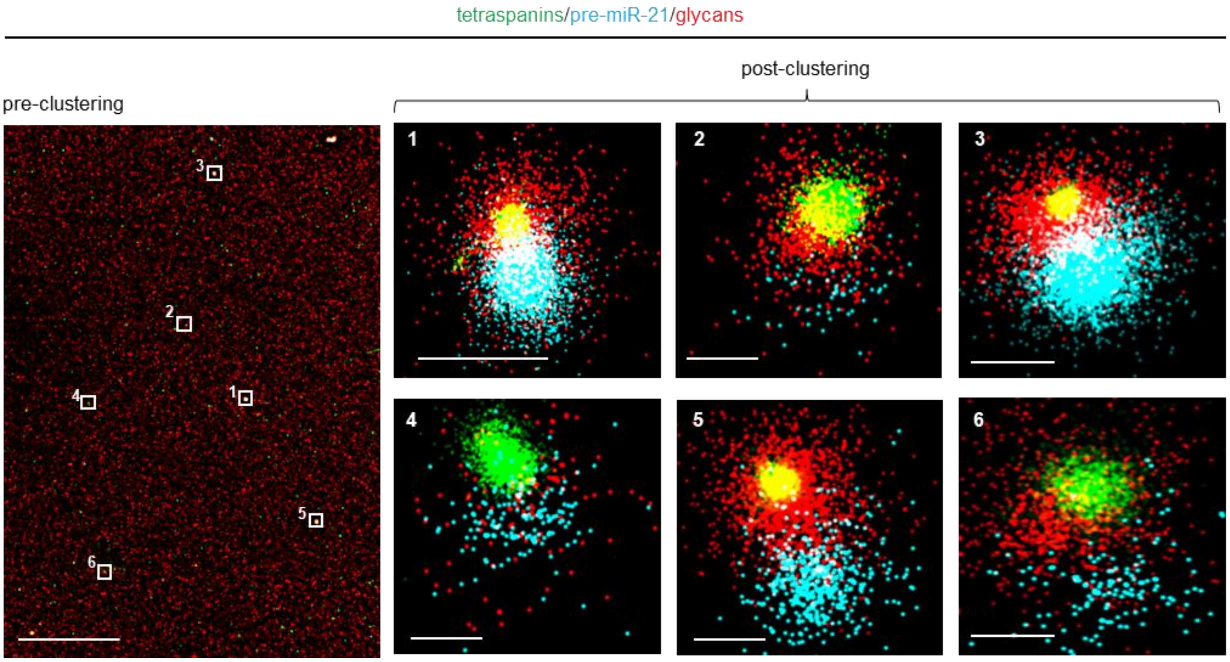

Figure S7. Super-resolution dSTORM images of exosomes isolated from human keratinocytes conditioned media showing localization of tetraspanin markers (CD9, CD63, CD81) on exosomes (green), with MB of pre-miR-21 (cyan) and Aminooxy labeled glycans (red). Scale, 10 μm. Zoomed images of a single exosome with tetraspanins, pre-miR-21, and glycans after clustering in CODI software. Scale, 100 nm.

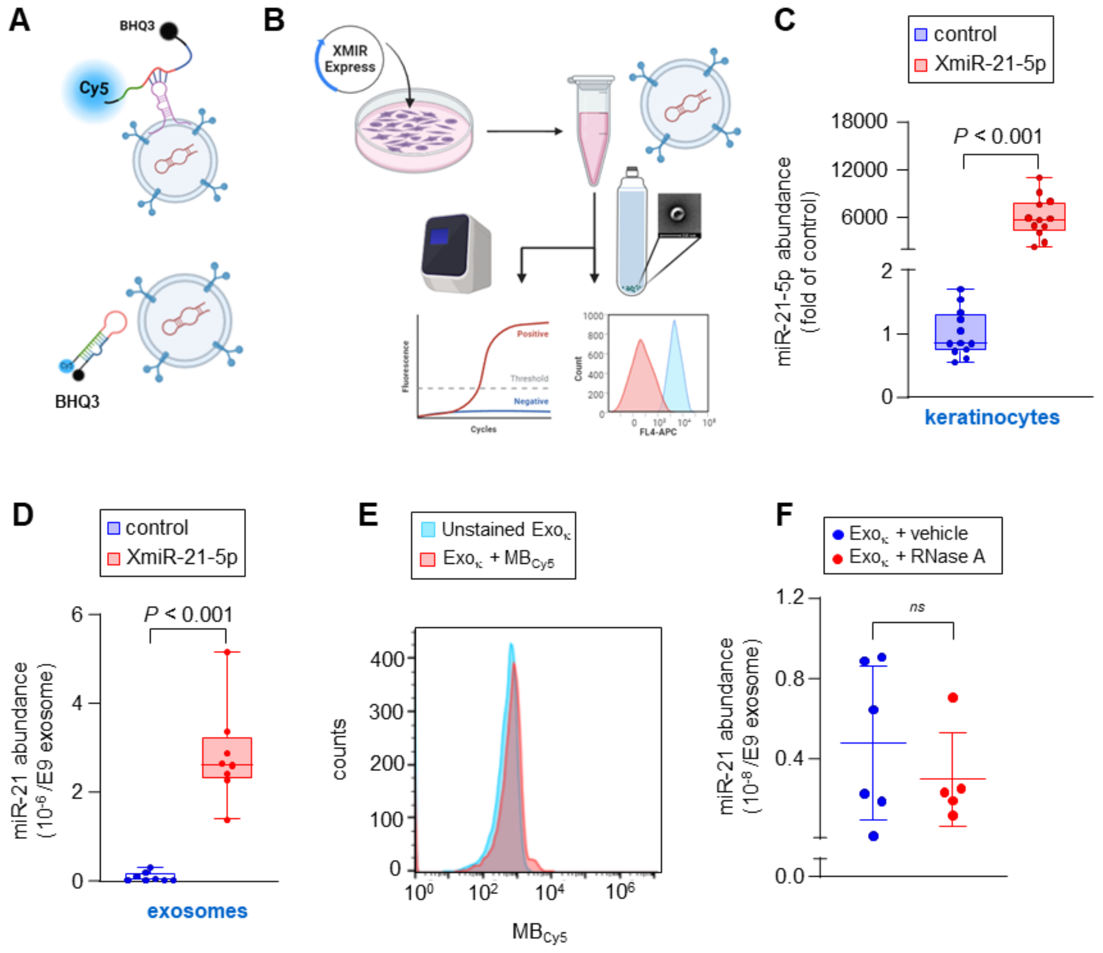

Figure S8. Validation of molecular beacon (MB). (A) Schematics showing the binding of MB with pre-miR-21 only when present on the exosome surface. Pre-miR-21 encapsulated within exosomes will not show any Cy5 signals. (B) Schematics showing transfection of XmiR-21-5p (chemically synthesized, double-stranded miR-21-5p that mimics mature endogenous miR21-5p after transfection and tagged with XMotif that targets exosomes for packaging into cells to human keratinocytes, followed by analysis of miR-21-5p by RT-qPCR and shift in Cy5 fluorescence of MB using beads flow cytometry. Abundance of miR-21-5p in (C) human keratinocytes and (D) in exosomes 48 h after transfection with XmiR-21-5p. (n=8-12). Data in (C) and (D) are shown as mean ± SEM and analyzed by Student's t-test. Data in (C) and (D) were reproduced with permission from Zhou et al., *ACS Nano 2020, 14, 10, 12732–12748*. Copyright (2020) American

Chemical Society. (E) No significant shift in Cy5 florescence was observed post XmiR-21-5p using bead flow cytometry. (F) Abundance of miR-21-5p in exosomes 48 h after transfection with XmiR-21-5p followed by either RNase A or vehicle treatment. (n=6). Data in (F) is shown as mean ± SD and analyzed by the Mann-Whitney U non-parametric test. Figure S8A and B were created with BioRender.com.

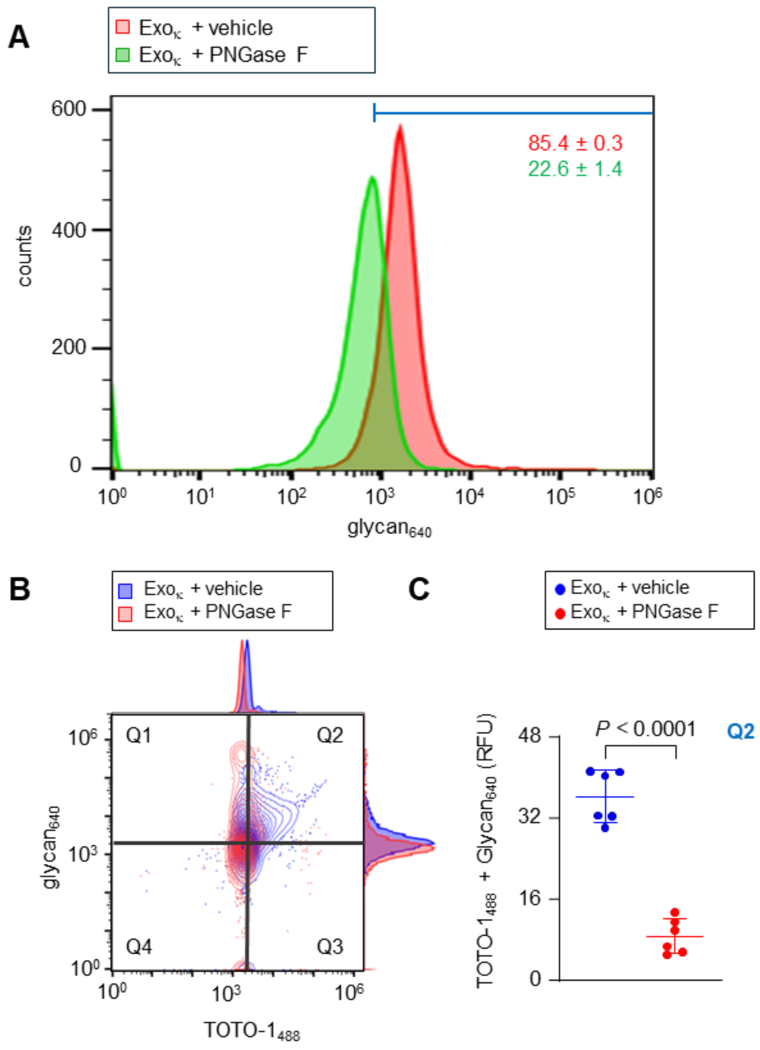

Figure S9. (A) Beads flow cytometry showing a significant decrease in glycan intensity of Exo$_\kappa$ with PNGase F treatment. Data shown as mean ± SD. (B) Representative beads flow cytometry scatter plot showing TOTO-1 and aminooxy fluorescence of Exo$_\kappa$ treated with either vehicle or PNGase F. (C) Quantification of glycoRNAs after treatment with either vehicle or PNGase F. (n=6). Data in (C) is shown as mean ± SD and analyzed by Student's t-test.

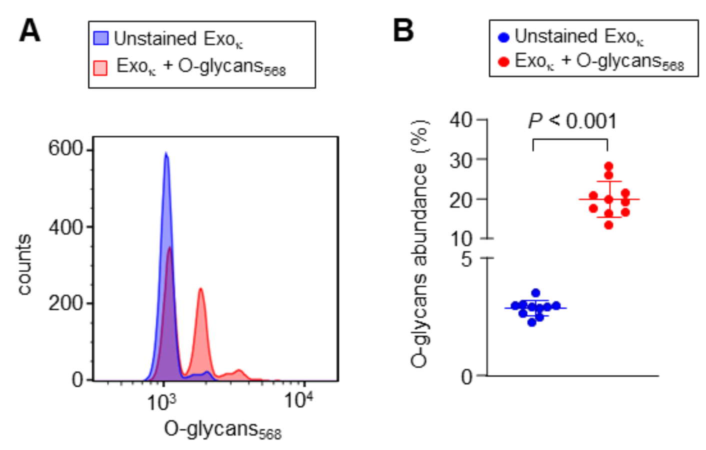

Figure S10. Beads flow cytometric analysis of Exoκ conjugated with super magnetic dynabeads™ functionalized with CD63, CD9, and CD81, showing the presence of *O*-glycans on Exoκ surface. (A) The histogram demonstrates the shift in PE fluorescence. The mean percentage of beads with exosomes showing PE fluorescence was plotted graphically. (B) Quantification of *O*-glycans upon enzymatic labeling. (n=10). Data in (B) is shown as mean ± SD and analyzed by Student's t-test.

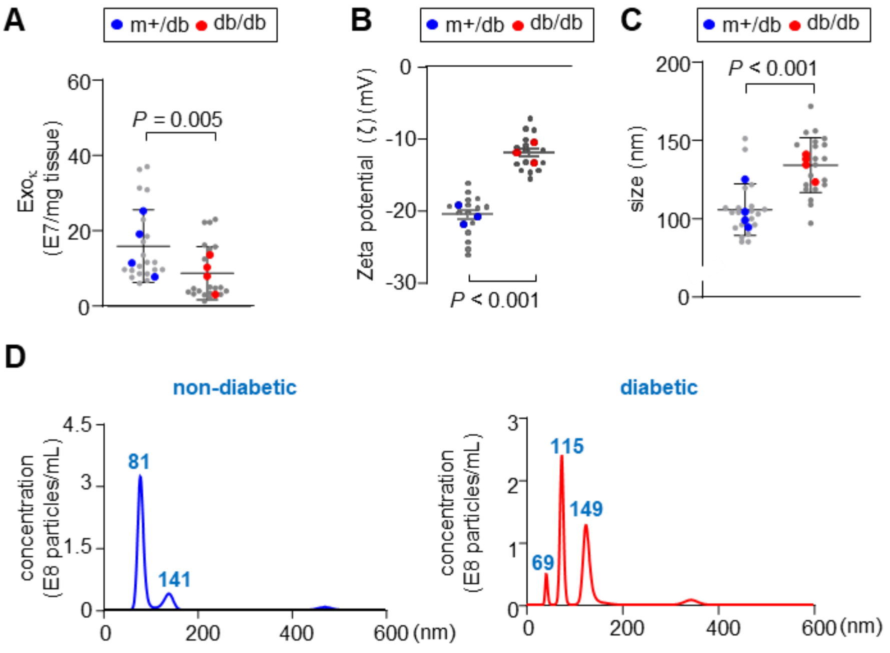

Figure S11. Characterization of murine Exo$_\kappa$. (A) Quantification of Exo$_\kappa$ isolated from D2 WE tissue of non-diabetic (m+/db) mice and diabetic (db/db) mice. Each grey dot corresponds to one technical replicate, and the blue and red dots correspond to the mean of each biological replicate. (n=4). (B) Zeta potentials of Exo$_\kappa$ at physiological pH (pH-7.4). Each grey dot corresponds to one technical replicate, and the blue and red dots correspond to the mean of each biological replicate. (n=3). (C-D) Quantification of Exo$_\kappa$ size (C) and histogram of size distribution (D) from representative NTA images and of Exo$_\kappa$ isolated from D2 WE tissue of non-diabetic (m+/db) mice and diabetic (db/db) mice. Each grey dot corresponds to one technical replicate, and the blue and red dots correspond to the mean of each biological replicate. (n=4). Data in (A), (B), and (C) are shown as mean ± SD and analyzed by Student's t-test.

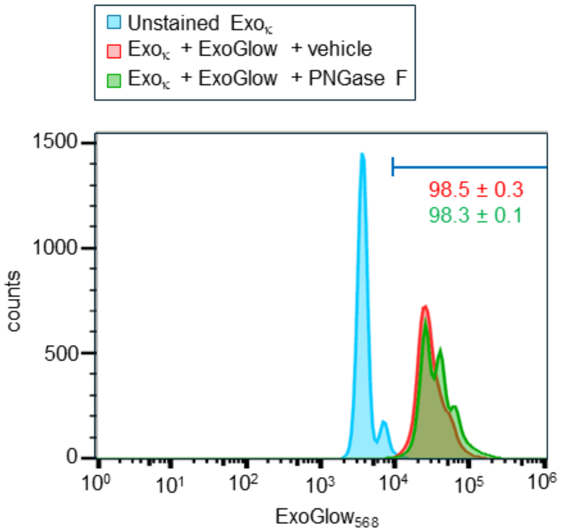

Figure S12. Beads flow cytometry showing PNGase F treatment does not affect ExoGlow fluorescence of Exo$_\kappa$.

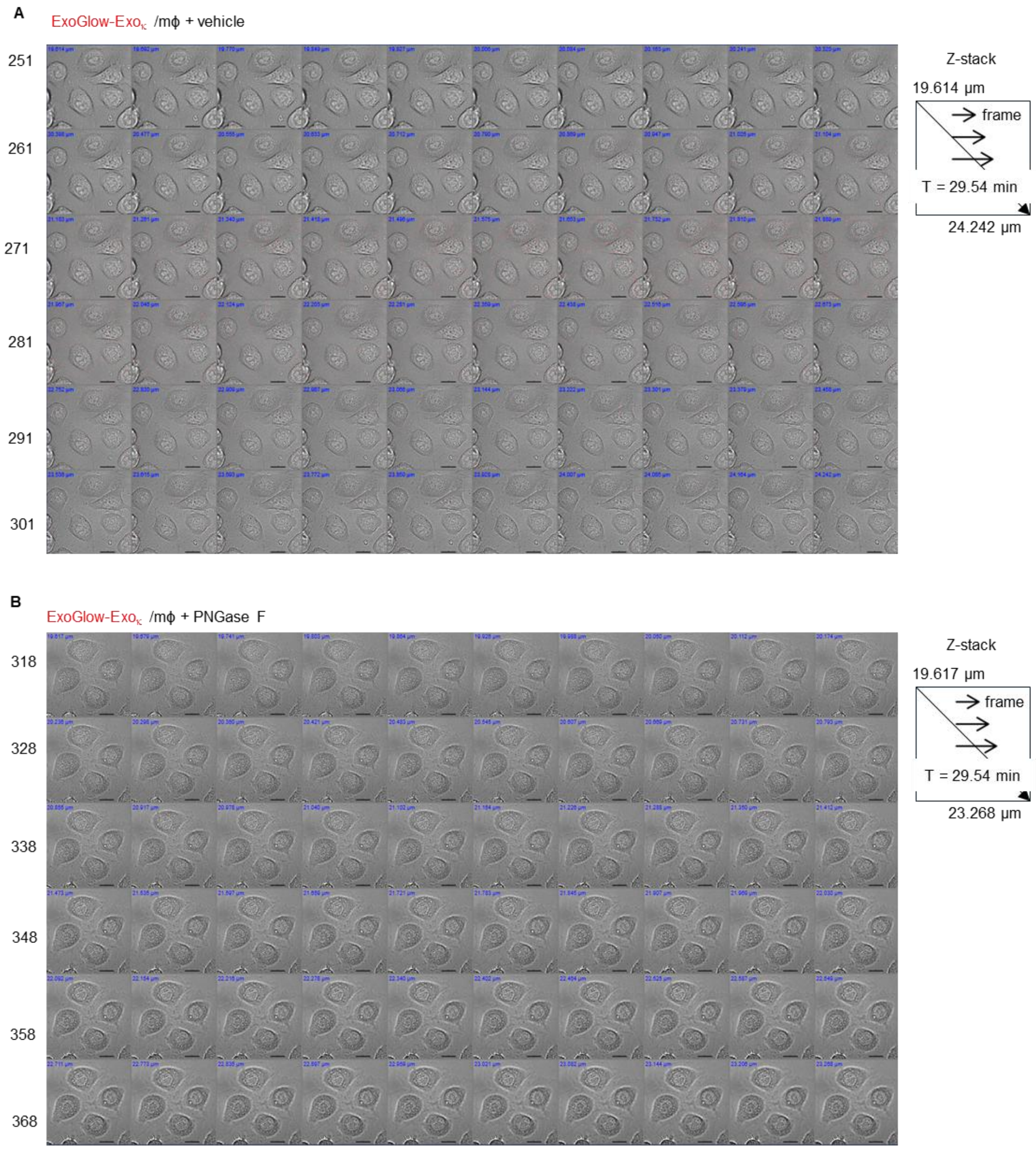

Figure S13. Z-stack image of exosome uptake assay by mϕ. Z-stack images of proinflammatory mΦ after incubation with ExoGlow labeled Exo$_{\kappa}$ without (A) and with PNGase F treatment (B) at the end of live cell imaging (related to Fig. 4A). Scale, 10 µm. The schematic on the right shows the direction of the tile images with increasing depth at the end of the uptake assay. T= 29.54 min.

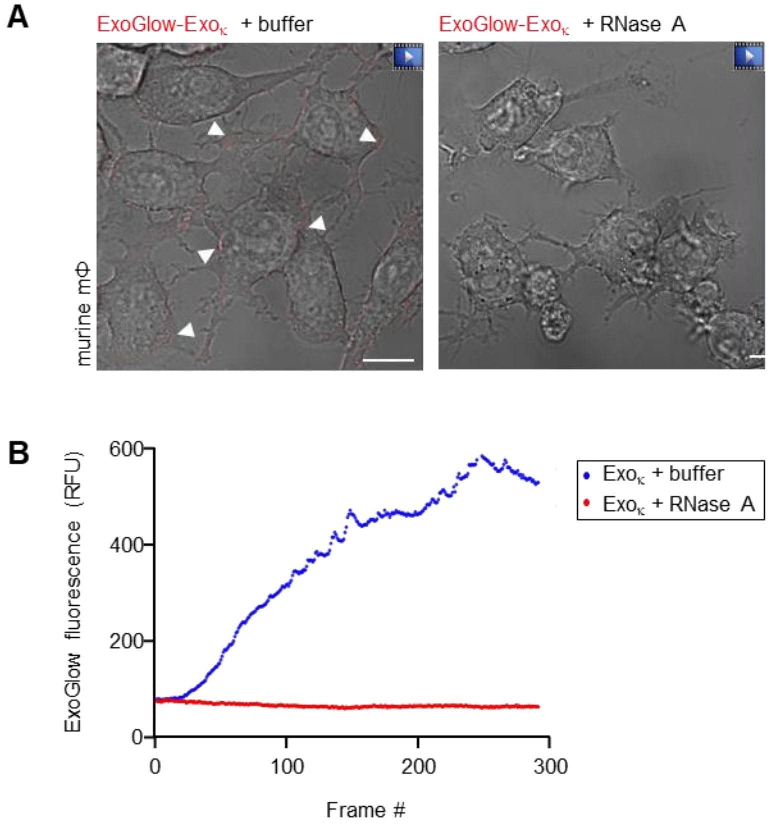

Figure S14. (A) Live-cell confocal images showing compromised uptake of Exo$_{\kappa}$ by proinflammatory macrophages following deletion of RNA/glycoRNA using RNase A treatment. Scale, 10 μm. Both Exo$_{\kappa}$ treated with and without RNase A were stained with ExoGlow before imaging. [movie icon] Indicate movies in the supplement. (B) Quantification of ExoGlow relative fluorescence intensity with time.

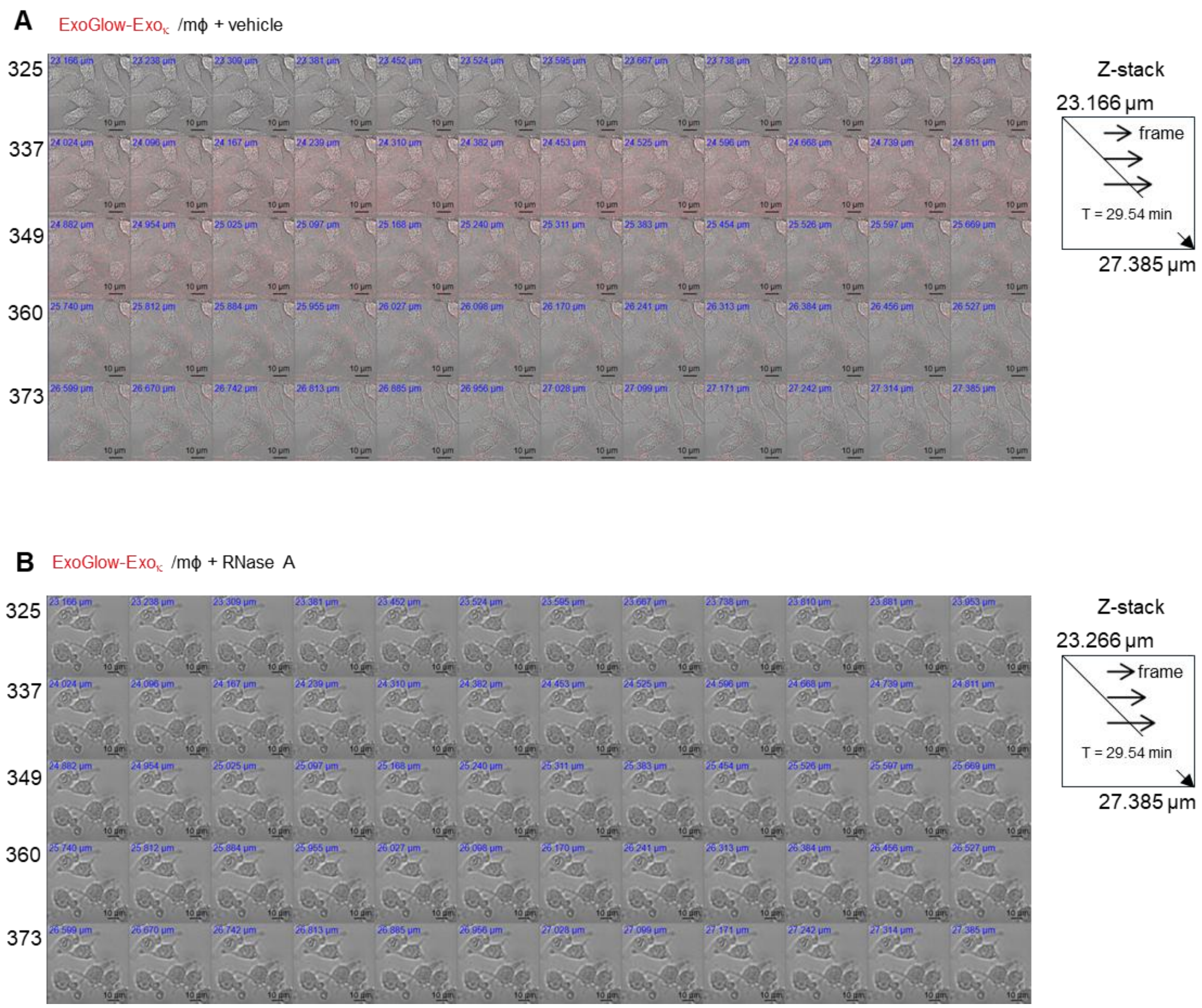

Figure S15. Z-stack image of exosome uptake assay by mϕ. Z-stack images of proinflammatory mΦ after incubation with ExoGlow labeled Exo_k without (A) and with RNase A treatment (B) at the end of live cell imaging (related to Fig. S14). Scale, 10 µm. The schematic on the right shows the direction of the tile images with increasing depth at the end of the uptake assay. T= 29.54 min.

Supporting Information

A

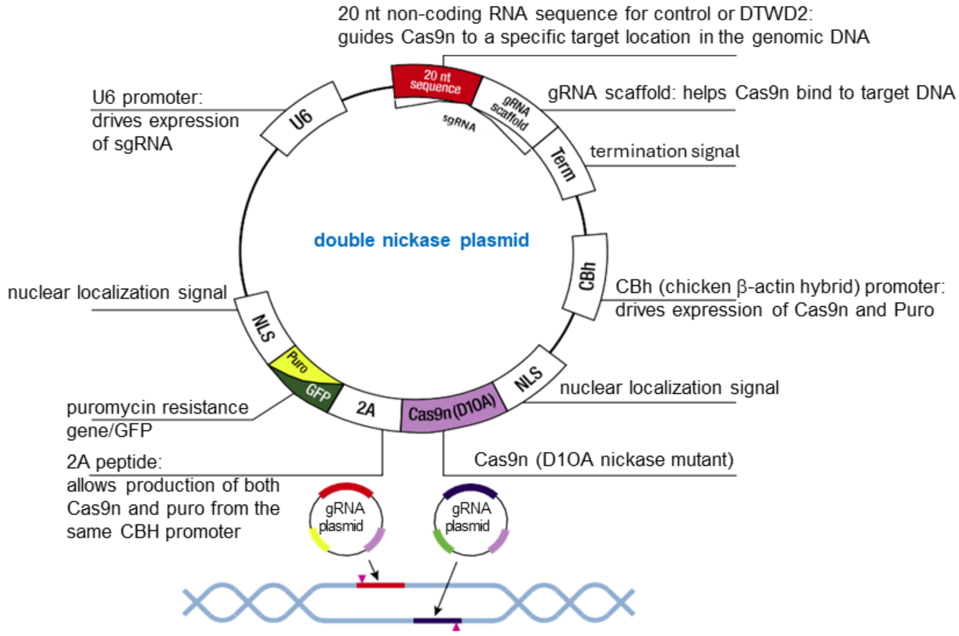

B

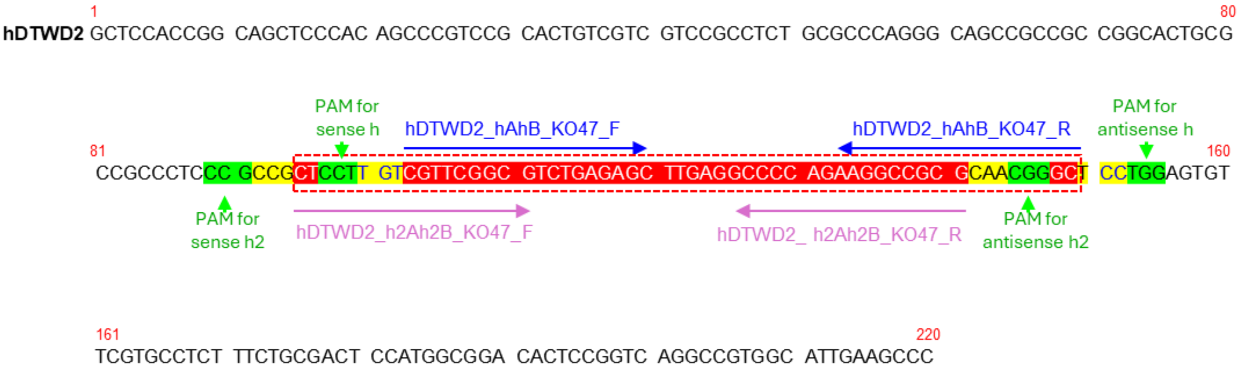

C

S/ No.	Primer Name	Primer Sequence (5' → 3')	Experiment
1	hDTWD2_hAhB_KO47_F	CGTTCGGCGTCTGAGAG	Gap RT-QPCR
2	hDTWD2_hAhB_KO47_R	GCCCGTTGCGCGGCCTT	Gap RT-QPCR
3	hDTWD2_h2Ah2B_KO47_F	CTCCTTGTCGTTCGGCG	Gap RT-QPCR
4	hDTWD2_h2Ah2B_KO47_R	CGCGGCCTTCTGGGGCCT	Gap RT-QPCR
5	hDTWD2_F	ATGGAGTCGCAGAAAGAGGC	RT-QPCR
6	hDTWD2_R	CTCCTTGTCGTTCGGCGT	RT-QPCR

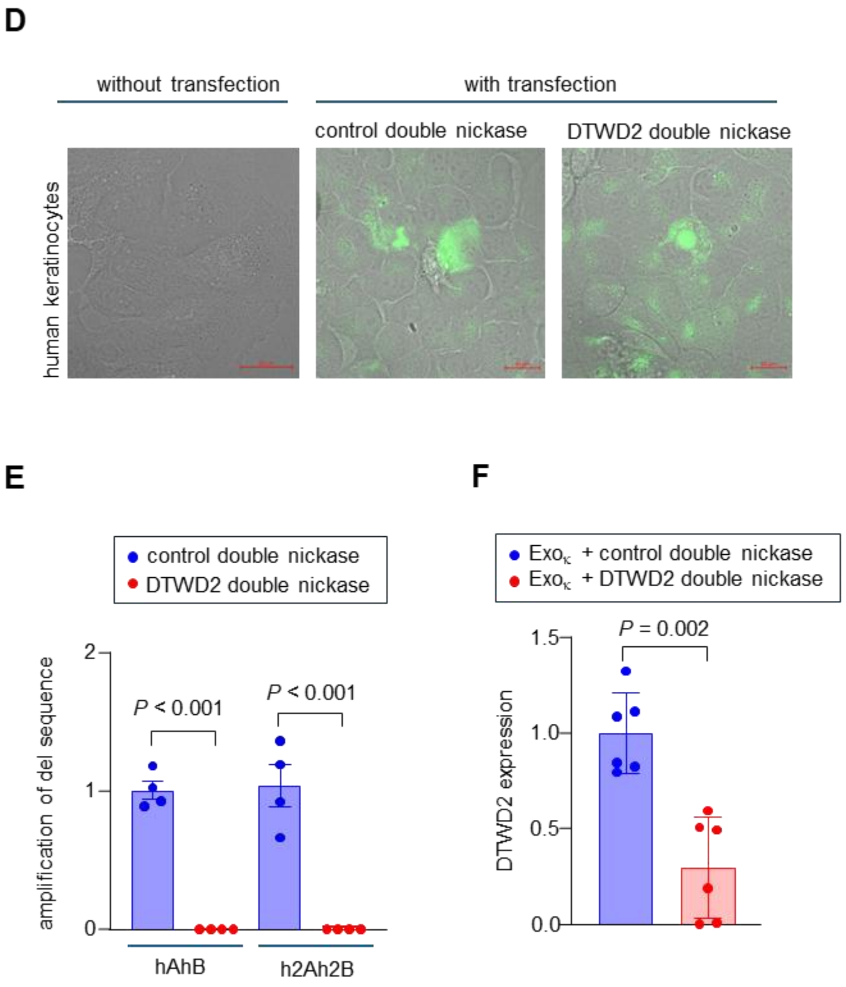

Figure S16. (A) Schematic showing the plasmid design. (B) The hDTWD gene sequence showing the Protospacer Adjacent Motif (PAM) immediately flanking the CRISPR target site that is required for Cas nuclease binding and cleavage sequence shown with the red dashed box, and the regions from where the primers were designed to check genomic deletion in human keratinocytes. (C) The primer sequences used for Gap RT-qPCR from genomic DNA and hDTWD2 expression from total RNA in human keratinocytes. (D) Representative confocal image showing transfection efficiency following delivery of the control and DTWD2 double nickase plasmids. (E) Unlike the control plasmid, no amplification was observed in the DTWD2 double nickase plasmid-transfected group. (F) DTWD2 knockout efficiency was validated using RT-qPCR. Data shown as mean ± SD

and analyzed by Student's t test (E) and Mann-Whitney U non-parametric test in (F). (n=4 for E; 6 for F)

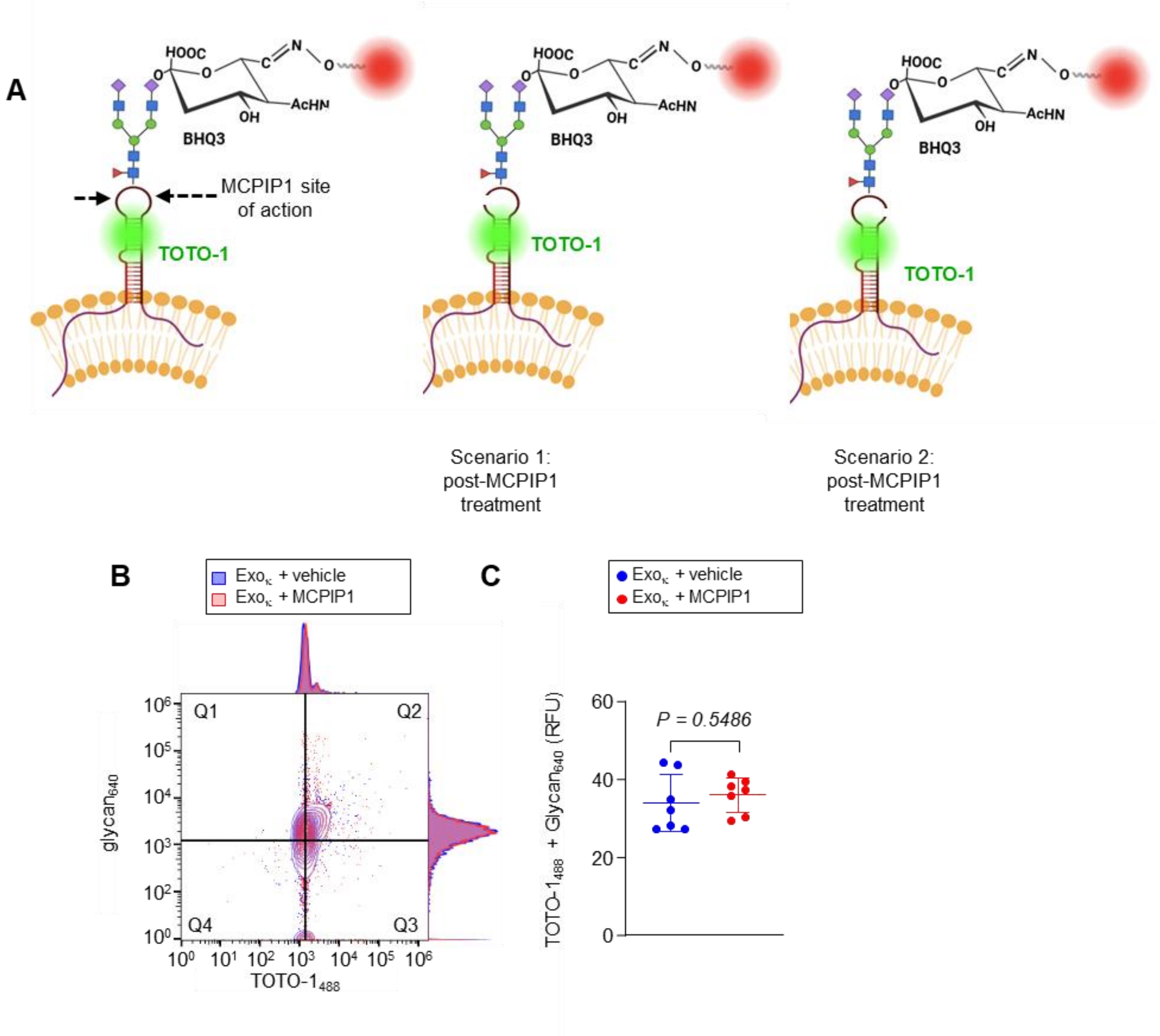

Figure S17. (A) Schematic showing the site of cleavage by endoribonuclease MCPIP1 and the probable scenario by which the glycoRNA signal can be affected. (B) Representative beads flow cytometry scatter plot showing TOTO-1 and aminooxy fluorescence of Exo$_\kappa$ treated with either

vehicle or MCPIP1. (C) Quantification of glycoRNAs after treatment with either vehicle or MCPIP1 (n=7). Data in (C) is shown as mean ± SD and analyzed by Student's t-test.

References

1. Ghatak, S.; Khanna, S.; Roy, S.; Thirunavukkarasu, M.; Pradeep, S. R.; Wulff, B. C.; El Masry, M. S.; Sharma, A.; Palakurti, R.; Ghosh, N.; Xuan, Y.; Wilgus, T. A.; Maulik, N.; Yoder, M. C.; Sen, C. K., Driving adult tissue repair via re-engagement of a pathway required for fetal healing. *Mol Ther* **2023,** *31* (2), 454-470.

2. Singh, K.; Rustagi, Y.; Abouhashem, A. S.; Tabasum, S.; Verma, P.; Hernandez, E.; Pal, D.; Khona, D. K.; Mohanty, S. K.; Kumar, M.; Srivastava, R.; Guda, P. R.; Verma, S. S.; Mahajan, S.; Killian, J. A.; Walker, L. A.; Ghatak, S.; Mathew-Steiner, S. S.; Wanczyk, K. E.; Liu, S.; Wan, J.; Yan, P.; Bundschuh, R.; Khanna, S.; Gordillo, G. M.; Murphy, M. P.; Roy, S.; Sen, C. K., Genome-wide DNA hypermethylation opposes healing in patients with chronic wounds by impairing epithelial-mesenchymal transition. *J Clin Invest* **2022,** *132* (17).

3. Guda, P. R.; Sharma, A.; Anthony, A. J.; ElMasry, M. S.; Couse, A. D.; Ghatak, P. D.; Das, A.; Timsina, L.; Trinidad, J. C.; Roy, S.; Clemmer, D. E.; Sen, C. K.; Ghatak, S., Nanoscopic and Functional Characterization of Keratinocyte-Originating Exosomes in the Wound Fluid of Non-Diabetic and Diabetic Chronic Wound Patients. *Nano Today* **2023,** *52*.

4. Zhou, X.; Brown, B. A.; Siegel, A. P.; El Masry, M. S.; Zeng, X.; Song, W.; Das, A.; Khandelwal, P.; Clark, A.; Singh, K.; Guda, P. R.; Gorain, M.; Timsina, L.; Xuan, Y.; Jacobson, S. C.; Novotny, M. V.; Roy, S.; Agarwal, M.; Lee, R. J.; Sen, C. K.; Clemmer, D. E.; Ghatak, S., Exosome-Mediated Crosstalk between Keratinocytes and Macrophages in Cutaneous Wound Healing. *ACS Nano* **2020,** *14* (10), 12732-12748.

5. Gallego-Perez, D.; Pal, D.; Ghatak, S.; Malkoc, V.; Higuita-Castro, N.; Gnyawali, S.; Chang, L.; Liao, W. C.; Shi, J.; Sinha, M.; Singh, K.; Steen, E.; Sunyecz, A.; Stewart, R.; Moore, J.; Ziebro, T.; Northcutt, R. G.; Homsy, M.; Bertani, P.; Lu, W.; Roy, S.; Khanna, S.; Rink, C.; Sundaresan, V. B.; Otero, J. J.; Lee, L. J.; Sen, C. K., Topical tissue nano-transfection mediates non-viral stroma reprogramming and rescue. *Nat Nanotechnol* **2017,** *12* (10), 974-979.

6. Li, Z.; Xuan, Y.; Ghatak, S.; Guda, P. R.; Roy, S.; Sen, C. K., Modeling the gene delivery process of the needle array-based tissue nanotransfection. *Nano Res* **2022,** *15* (4), 3409-3421.

7. Xuan, Y.; Ghatak, S.; Clark, A.; Li, Z.; Khanna, S.; Pak, D.; Agarwal, M.; Roy, S.; Duda, P.; Sen, C. K., Fabrication and use of silicon hollow-needle arrays to achieve tissue nanotransfection in mouse tissue in vivo. *Nat Protoc* **2021,** *16* (12), 5707-5738.

8. Brown, B. A.; Guda, P. R.; Zeng, X.; Anthony, A.; Couse, A.; Barnes, L. F.; Sharon, E. M.; Trinidad, J. C.; Sen, C. K.; Jarrold, M. F.; Ghatak, S.; Clemmer, D. E., Analysis of Keratinocytic Exosomes from Diabetic and Nondiabetic Mice by Charge Detection Mass Spectrometry. *Anal Chem* **2022,** *94* (25), 8909-8918.

9. Roy, S.; Sen, C. K.; Ghatak, S.; Higuita-Castro, N.; Palakurti, R.; Nalluri, N.; Clark, A.; Stewart, R.; Gallego-Perez, D.; Prater, D. N.; Khanna, S., Neurogenic tissue Nanotransfection in the Management of Cutaneous Diabetic Polyneuropathy. *Nanomedicine* **2020**, 102220.

10. Pal, D.; Ghatak, S.; Singh, K.; Abouhashem, A. S.; Kumar, M.; El Masry, M. S.; Mohanty, S. K.; Palakurti, R.; Rustagi, Y.; Tabasum, S.; Khona, D. K.; Khanna, S.; Kacar, S.; Srivastava, R.; Bhasme, P.; Verma, S. S.; Hernandez, E.; Sharma, A.; Reese, D.; Verma, P.; Ghosh, N.; Gorain, M.; Wan, J.; Liu, S.; Liu, Y.; Castro, N. H.; Gnyawali, S. C.; Lawrence, W.; Moore, J.; Perez, D. G.; Roy, S.; Yoder, M. C.; Sen, C. K., Identification of a physiologic vasculogenic fibroblast state to achieve tissue repair. *Nat Commun* **2023,** *14* (1), 1129.

11. Li, Z.; Xuan, Y.; Ghatak, S.; Guda, P. R.; Roy, S.; Sen, C. K., Modeling the gene delivery process of the needle array-based tissue nanotransfection. *Nano Research* **2022,** *15* (4), 3409-3421.

12. Clark, A.; Ghatak, S.; Guda, P. R.; El Masry, M. S.; Xuan, Y.; Sato, A. Y.; Bellido, T.; Sen, C. K., Myogenic tissue nanotransfection improves muscle torque recovery following volumetric muscle loss. *NPJ Regen Med* **2022,** *7* (1), 63.

13. Ghatak, S.; Khanna, S.; Roy, S.; Thirunavukkarasu, M.; Pradeep, S. R.; Wulff, B. C.; ElMasry, M. S.; Sharma, A.; Palakurti, R.; Ghosh, N.; Xuan, Y.; Wilgus, T. A.; Maulik, N.; Yoder, M. C.; Sen, C. K., Driving adult tissue repair via re-engagement of a pathway required for fetal healing. *Mol Ther* **2022**.

14. Hemberger, H.; Chai, P.; Lebedenko, C.; Caldwell, R.; George, B.; Flynn, R., *Rapid and sensitive detection of native glycoRNAs*. 2023.

15. Deng, B.; Ghatak, S.; Sarkar, S.; Singh, K.; Das Ghatak, P.; Mathew-Steiner, S. S.; Roy, S.; Khanna, S.; Wozniak, D. J.; McComb, D. W.; Sen, C. K., Novel Bacterial Diversity and Fragmented eDNA Identified in Hyperbiofilm-Forming Pseudomonas aeruginosa Rugose Small Colony Variant. *iScience* **2020,** *23* (2), 100827.

16. Gordillo, G. M.; Guda, P. R.; Singh, K.; Biswas, A.; Abouhashem, A. S.; Rustagi, Y.; Sen, A.; Kumar, M.; Das, A.; Ghatak, S.; Khanna, S.; Sen, C. K.; Roy, S., Tissue nanotransfection causes tumor regression by its effect on nanovesicle cargo that alters microenvironmental macrophage state. *Mol Ther* **2023,** *31* (5), 1402-1417.

17. Ghatak, S.; Li, J.; Chan, Y. C.; Gnyawali, S. C.; Steen, E.; Yung, B. C.; Khanna, S.; Roy, S.; Lee, R. J.; Sen, C. K., AntihypoxamiR functionalized gramicidin lipid nanoparticles rescue against ischemic memory improving cutaneous wound healing. *Nanomedicine* **2016,** *12* (7), 1827-1831.

18. Das, A.; Ganesh, K.; Khanna, S.; Sen, C. K.; Roy, S., Engulfment of apoptotic cells by macrophages: a role of microRNA-21 in the resolution of wound inflammation. *Journal of immunology* **2014,** *192* (3), 1120-9.

19. Clos-Sansalvador, M.; Garcia, S. G.; Morón-Font, M.; Williams, C.; Reichardt, N. C.; Falcón-Pérez, J. M.; Bayes-Genis, A.; Roura, S.; Franquesa, M.; Monguió-Tortajada, M.; Borràs, F. E., N-Glycans in Immortalized Mesenchymal Stromal Cell-Derived Extracellular Vesicles Are Critical for EV-Cell Interaction and Functional Activation of Endothelial Cells. *Int J Mol Sci* **2022,** *23* (17).

20. Ma, Y.; Guo, W.; Mou, Q.; Shao, X.; Lyu, M.; Garcia, V.; Kong, L.; Lewis, W.; Ward, C.; Yang, Z.; Pan, X.; Yi, S. S.; Lu, Y., Spatial imaging of glycoRNA in single cells with ARPLA. *Nat Biotechnol* **2024,** *42* (4), 608-616.
